# Evolution and roles of cytokinin genes in angiosperms 2: Do ancient *CKX*s play housekeeping roles while non-ancient *CKX*s play regulatory roles?

**DOI:** 10.1038/s41438-020-0246-z

**Published:** 2020-03-01

**Authors:** Xiaojing Wang, Jing Ding, Shanshan Lin, Decai Liu, Tingting Gu, Han Wu, Robert N. Trigiano, Richard McAvoy, Jinling Huang, Yi Li

**Affiliations:** 1 State Key Laboratory of Crop Genetics and Germplasm Enhancement and College of Horticulture, Nanjing Agricultural University, Nanjing, P. R. China; 2 Department of Entomology and Plant Pathology, University of Tennessee, Knoxville, TN 37996-4560 USA; 3 Department of Plant Science and Landscape Architecture, University of Connecticut, Storrs, CT 06269 USA; 4 State Key Laboratory of Crop Stress Adaptation and Improvement, Key Laboratory of Plant Stress Biology, School of Life Sciences, Henan University, Kaifeng, China; 5 Department of Biology, East Carolina University, Greenville, NC 27858 USA

**Keywords:** Cytokinin, Plant evolution

## Abstract

Cytokinin oxidase/dehydrogenase (CKX) is a key enzyme responsible for the degradation of endogenous cytokinins. However, the origins and roles of *CKX* genes in angiosperm evolution remain unclear. Based on comprehensive bioinformatic and transgenic plant analyses, we demonstrate that the *CKX*s of land plants most likely originated from an ancient chlamydial endosymbiont during primary endosymbiosis. We refer to the *CKX*s retaining evolutionarily ancient characteristics as “ancient *CKX*s” and those that have expanded and functionally diverged in angiosperms as “non-ancient *CKX*s”. We show that the expression of some non-ancient *CKX*s is rapidly inducible within 15 min upon the dehydration of *Arabidopsis*, while the ancient *CKX* (*AtCKX7*) is not drought responsive. Tobacco plants overexpressing a non-ancient *CKX* display improved oxidative and drought tolerance and root growth. Previous mutant studies have shown that non-ancient *CKX*s regulate organ development, particularly that of flowers. Furthermore, ancient CKXs preferentially degrade *cis*-zeatin (*c*Z)-type cytokinins, while non-ancient CKXs preferentially target *N*^6^-(Δ^2^-isopentenyl) adenines (iPs) and *trans*-zeatins (*t*Zs). Based on the results of this work, an accompanying study (Wang et al. 10.1038/s41438-019-0211-x) and previous studies, we hypothesize that non-ancient CKXs and their preferred substrates of iP/*t*Z-type cytokinins regulate angiosperm organ development and environmental stress responses, while ancient CKXs and their preferred substrates of *c*Zs play a housekeeping role, which echoes the conclusions and hypothesis described in the accompanying report (Wang, X. et al. Evolution and roles of cytokinin genes in angiosperms 1: Doancient IPTs play housekeeping while non-ancient IPTs play regulatory roles? *Hortic Res* **7**, (2020). 10.1038/s41438-019-0211-x).

## Introduction

Cytokinins are a major class of hormones that regulate many developmental processes in plants, including cell division, shoot and root growth, and vascular and gametophyte development^1^. Cytokinins also play important roles in the responses of plants to biotic and abiotic stresses. Natural cytokinins are *N*^6^-substituted adenine derivatives, and their most common forms in plants are *N*^6^-(Δ^2^-isopentenyl) adenines (iPs), *trans*-zeatins (*t*Zs), and *cis*-zeatins (*c*Zs). These cytokinin forms exhibit different levels of physiological activity and are differentially distributed among land plant lineages and in various tissues or stages of angiosperms^2^.

Angiosperms exhibit an efficient system for controlling the homeostasis of endogenous cytokinins in different organs and tissues. The major enzymes involved in the regulation of cytokinin content are isopentenyltransferases (IPTs) and “LONELY GUY” (LOGs, named by Kurakawa et al.^3^) cytokinin-specific phosphoribohydrolases for biosynthesis, cytokinin glucosyltransferases for conjugation, and cytokinin oxidases/dehydrogenases (CKXs) for irreversible degradation^4^. The *ATP/ADP-* and *tRNA-IPT* genes encode the main enzymes responsible for the biosynthesis of *t*Z- and *c*Z-type cytokinins in angiosperms, respectively^5^. IPT activity and active cytokinins have been detected in various algae^6,7^, suggesting that cytokinin biosynthesis already existed before plants were established on land. However, only *tRNA-IPT* genes have been identified in seedless plants^8^, and *ATP/ADP*-*IPT* genes are proposed to have arisen during the evolution of angiosperms, probably due to the need for higher levels of cytokinins^4^.

CKXs play an essential role in decreasing endogenous cytokinin content. Cytokinin glucosyltransferases can also reduce active cytokinins by conjugation, but their effects are mostly reversible, and changes in their gene expression do not always lead to variations in active cytokinin levels or phenotypes^1,9^. In contrast, CKXs are the only known proteins that specifically degrade cytokinins, including their ribosides and some glucosides^10^. The manipulation of *CKX* gene expression can substantially alter the levels of active cytokinins, resulting in physiological or developmental changes^11–13^. CKX isoforms show differences in their subcellular localization, substrate preference and other properties^10^. The overexpression of individual *Arabidopsis CKX* (*AtCKX*) genes results in various root phenotypes^11,13^, suggesting that CKX isoforms play different roles in plant organ development.

*CKX* genes have also been shown to be involved in plant tolerance to abiotic stresses^12,14–16^. Carabelli et al.^17^ demonstrated that *AtCKX6* is critical for the growth arrest of the leaf primordium, which contributes to extension growth under shaded conditions. The constitutive or root-specific overexpression of *AtCKX1-4* in *Arabidopsis* and other species results in improved drought, salt and heat tolerance compared with wild-type plants^12,14,18^.

Despite the importance of *CKX* genes in angiosperms, their origin and evolution are poorly studied. In addition to land plants, CKX activity and homologous *CKX* sequences have been detected in bacteria such as *Rhodococcus fascians*^19^ but not in any algae. The plant *CKX* genes were once suggested to have been derived from cyanobacteria^20^, along with thousands of other nuclear genes transferred from plastids^21^. Furthermore, among the angiosperm *CKX* genes, using *Arabidopsis* as an example, *AtCKX7* has been proposed to be an evolutionarily ancient isoform based on the comparison of protein characteristics and the changes observed in *AtCKX*-overexpressing plants between different *AtCKX* members^13^. We refer to *AtCKX7* and its orthologs in other angiosperms as ancient *CKX*s. Our systematic survey and phylogenetic analysis of *CKX* genes from bacteria, archaea, and eukaryotes provide evidence supporting the hypothesis that all land plant *CKX* genes are derived from a single *CKX* with a chlamydial origin. We therefore refer to the genes of angiosperms that have expanded and functionally diverged from the ancient genes as non-ancient *CKX*s. We show that the non-ancient *CKX* genes are expressed in a tissue/organ-specific manner and exhibit rapid positive responses to dehydration and that their overexpression increases plant stress tolerance and root growth and development. On the other hand, transcriptome and qPCR analyses in several angiosperm species show that the expression of ancient *CKX*s is generally constitutive in tissues/organs and is non-stress responsive. Our findings provide important insights into the origin, evolution, and possible roles of *CKX* genes and give rise to the hypothesis that non-ancient *CKX*s and their preferred substrates, iPs and *t*Zs, play regulatory roles in organ development and stress tolerance in angiosperms, while ancient *CKX*s and their preferred substrates, *c*Zs, likely play a housekeeping role.

## Results

### *CKX* genes are largely restricted to land plants and bacteria

**Fig. 1:**
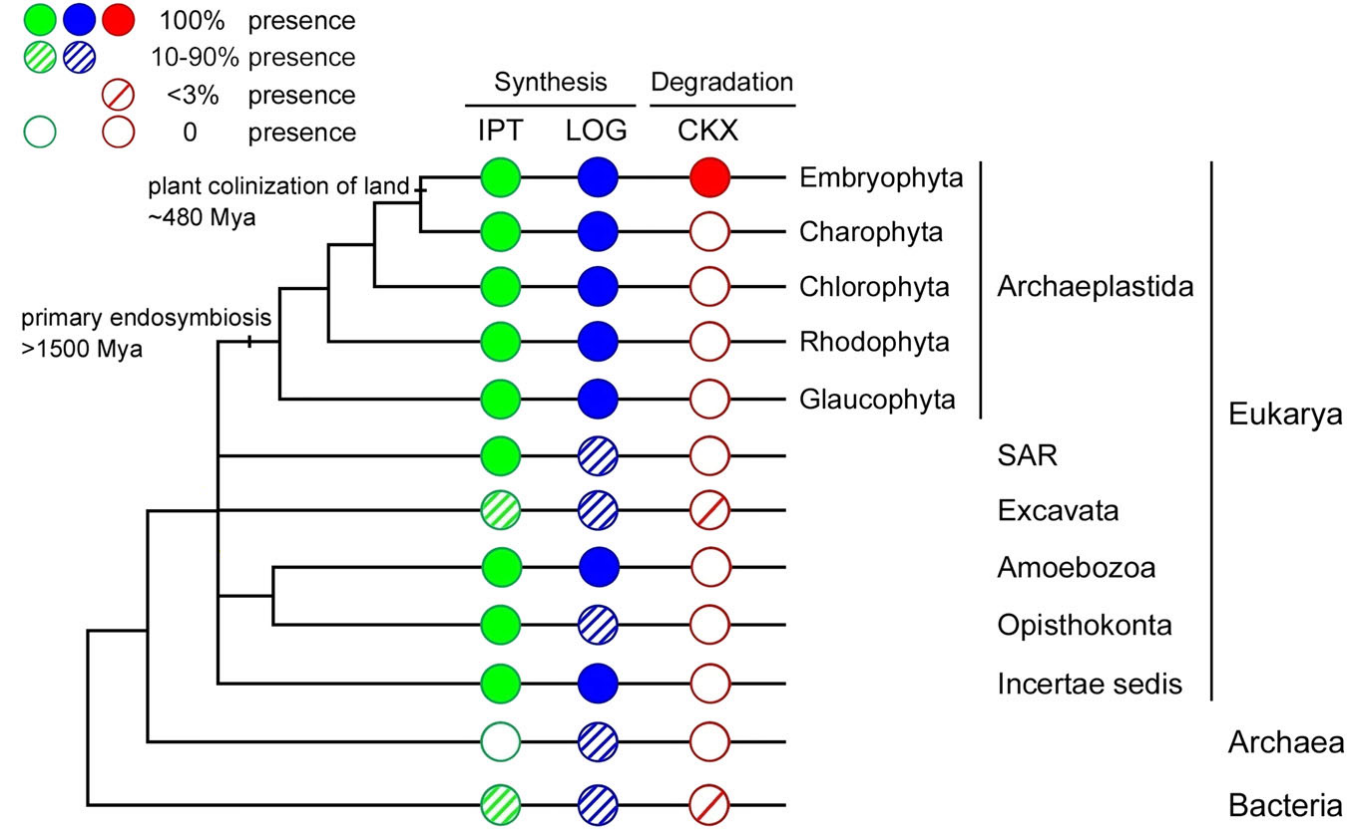
Distribution of cytokinin biosynthesis (*IPT* and *LOG*) and degradation (*CKX*) genes in living organisms, showing that *CKX* genes are uniquely restricted to land plants, Excavata and a small number of bacteria. Cytokinin biosynthesis and degradation genes and their homologs (*IPT*s, green; *LOG*s, blue; and *CKX*s, red circles) are present in all (100%, solid circles), a high percentage (10–90%, multiple-slashes filled circles), a low percentage (<3%, one-slash filled circles), or none (open circles) of the lineages within each taxon. Fifty complete genomes from representative lineages of bacteria, archaea, and eukaryotes (see Table S2) were sampled to estimate the percentages of the presence of *IPT* and *LOG* genes. Branch length is not proportional to evolutionary time.

*CKX* genes have been previously reported in land plants and bacteria but seldom in other organisms^4^. To systematically investigate the taxonomic distribution of *CKX* genes, we first conducted exhaustive HMMER, BLASTP, or TBLASTN searches for the Cytokin-bind domain (Pfam: PF09265), which is characteristic of CKX proteins, in organisms outside of land plants and bacteria. Only a single homolog, *NgrCKX1*, in the excavate species *Naegleria gruberi* (Fig. 1; Table S1), could be identified among these organisms. We could not detect any other *CKX* homologs in over 50 other excavate species whose complete genomes are publicly available.

We then searched the sequences of land plants and bacteria. *CKX* genes were identified in all available complete proteomes of land plants (Table S3). To compensate for the deficiency of complete proteomes from seedless land plants, we also searched the 1KP database, which contains transcriptomic sequences of many nonvascular plants (liverworts, mosses, and hornworts) and seedless vascular plants (lycophytes and monilophytes). At least one *CKX* gene was detected in 158 (94.6%) species across major lineages of 167 nonvascular and seedless vascular plants; no *CKX* homologs could be found in the remaining species, most likely because of their incomplete transcriptomic sequences (Table S4). As such, it is highly likely that *CKX* genes are ubiquitously present in all major lineages of land plants. On the other hand, homologous *CKX* sequences were identified in less than 2.5% of bacterial proteomes/genomes (240 out of more than 9,700) downloaded from the GenBank and JGI databases. The bacteria containing putative *CKX* genes predominantly come from Actinobacteria, Proteobacteria (alpha-, beta-, delta-, and gamma-) and Cyanobacteria, plus a few from Chlamydiae, Chloroflexi, and unclassified bacteria. No *CKX* homolog could be identified in other bacterial phyla. Collectively, the results of our survey demonstrate that *CKX* genes are ubiquitously present in land plants, one excavate species and a small percentage of bacteria, providing evidence that the *CKX* genes of land plants and bacteria are closely related, which is likely attributable to gene transfer between the two groups.

We also investigated the taxonomic distribution of cytokinin biosynthesis genes (*IPT*s and *LOG*s). Homologs of both *IPT* and *LOG* genes could be detected in a majority of the major lineages of bacteria and eukaryotes and several groups of archaea (Fig. 1; Table S2). Such a wide distribution strongly suggests that *IPT*s and *LOG*s have very ancient origins and are inherited among major taxa by conventional vertical descent, in sharp contrast to the *CKX* genes responsible for cytokinin degradation.

### Land plant *CKX* genes are likely derived from Chlamydiae

**Fig. 2:**
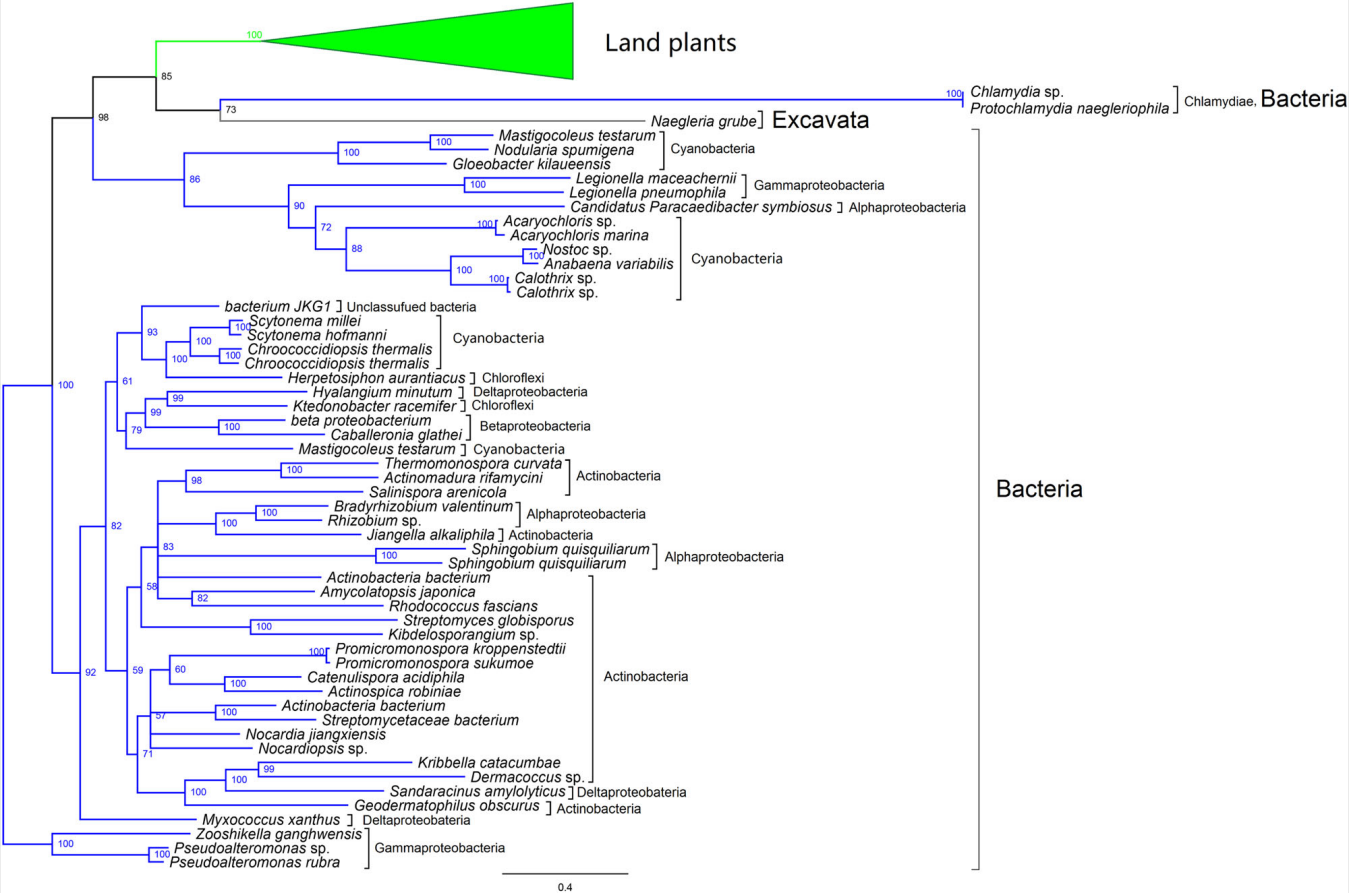
Phylogenetic analyses demonstrate a close relationship between land plant and chlamydial CKXs. The reduced MrBayes phylogeny (see Fig. S1 for the full tree topology) constructed from the two domain regions of the CKX proteins of six representative land plants, one excavate, and 49 selected bacteria (see Table S1) is shown. Support values (Bayesian posterior probabilities×100) greater than 50 are indicated at the nodes. Color coding: Green, land plants; blue, bacteria; gray, excavate.

We further performed phylogenetic analyses to understand the evolutionary relationships of the *CKX* genes. The phylogenies constructed from ML and Bayesian analyses provided similar relationships among land plant, bacterial and excavate CKXs (Figs. 2, S1 and S2). All land plant CKXs form a well-supported clade, which is a sister clade to a monophyletic group comprising chlamydial homologs and NgrCKX1. This large group of plant, chlamydial and *Naeglaria* sequences is in turn affiliated with cyanobacterial and proteobacterial homologs. Given the intimate physical association of Chlamydiae and *Naegleria*^22^, it is likely that *NgrCKX1* was acquired from Chlamydiae. The close relationship between land plant and chlamydial *CKX*s is reminiscent of other genes of chlamydial origin frequently reported in photosynthetic eukaryotes^23–25^. Therefore, land plant *CKX* genes may also be derived from Chlamydiae.

We compared the *CKX* sequence divergence between land plants, Chlamydiae, and other bacteria to assess whether the observed relationship of land plant and chlamydial *CKX* genes might be due to an artifact. The Ks value (synonymous nucleotide substitutions per synonymous site), which has been used as a proxy for divergence time between duplicates^26^, was calculated between each putative *CKX* gene pair sampled for the phylogenetic analysis. The results indicated that the average Ks value of the *CKX* pairs between land plants and Chlamydiae is lower than those between land plants and other bacterial groups (Table S5). In contrast, the highest Ka (nonsynonymous substitutions per nonsynonymous site) and Ka/Ks values are observed between land plants and Chlamydiae. These results suggest that the divergence of land plant and chlamydial *CKX*s occurred relatively late and that the chlamydial CKX protein sequences evolved faster than those of other bacteria. Due to these high Ka values, the amino acid similarities between the chlamydial and land plant CKXs are not high (approximately 25%). However, chlamydial CKXs contain the two key domains: the Cytokin-bind and FAD_binding_4 domains, and share core motifs with land plant CKXs (Fig. S3). Although Ks values may be affected by different evolutionary rates or become saturated over a long timescale, our Ka/Ks and sequence structure analyses suggest the closest relationship between land plant and chlamydial *CKX*s, thus indicating a chlamydial origin of land plant *CKX*s.

### Evolutionary patterns of *CKX* genes in angiosperms

We next investigated the evolution of the *CKX* genes in land plants after their transfer from Chlamydiae. Twenty-one species with available whole-genome sequences (Table S6) were sampled from all major lineages of land plants. A total of 184 *CKX* genes were identified. The number of *CKX* genes varied from two in the liverwort *Marchantia polymorpha* and the lycophyte *Selaginella moellendorffii* to 17 in the core eudicot *Glycine max* (Table S6), indicating the expansion of the *CKX* genes during land plant evolution.

**Fig. 3:**
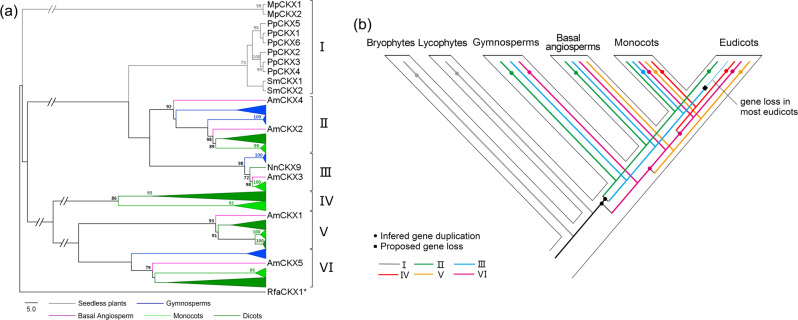
Phylogenetic analysis and evolutionary history of *CKX* genes in land plants. **a** Reduced PhyML phylogeny (see Fig. S4 for the full tree topology) of the CKX proteins from 21 representative land plants constructed based on the Cytokin-bind domain region. Bootstrap values greater than 70 are indicated at the nodes. The color coding for the plant lineages is indicated at the bottom. The abbreviations for the species names in front of the gene names are indicated in Table S6. **b** Inferred expansion history of the gene family in land plants. Inferred duplications and proposed losses are indicated with solid circles and black diamonds, respectively. Colored lines represent the respective groups in **a**, Fig. S4 and Table S6.

Phylogenetic analysis of land plant CKXs was performed using the actinobacterial *R. fascians* homolog as an outgroup (Fig. S4). In the phylogeny, the CKX sequences of seedless plants (including *M. polymorpha*, *Physcomitrella patens* and *S. moellendorffii*) clustered with their paralogs from the same species (Group I in Figs. 3a, S4 and Table S6), suggesting that the *CKX* gene may have been retained in a single copy before the divergence of seedless plants and seed plants (Fig. 3b). Seed plant CKXs can be divided into five major groups (Groups II-VI in Figs. 3a, S4 and Table S6). Among these groups, Groups II and III appear to be more closely related to Group I, consisting of the seedless plant CKX homologs, than to the other groups (Figs. 3, S4), although the bootstrap support for this relationship was modest. Motif composition analysis showed a closer relationship between Group I and Group II, rather than between Group I and Group III^27^. Furthermore, the ancient AtCKX7 isoform of *Arabidopsis*^13^ belongs to Group II. Biochemical and other characterizations of ZmCKXs in maize suggest that the Group II protein ZmCKX10 shows similar characteristics to AtCKX7 and that the ZmCKXs in Groups III-VI show distinct features^28,29^. Therefore, Group II is expected to be the only group containing ancient *CKX*s in angiosperms. Hereafter, we refer to this group as the ancient CKX group and to the other groups that contain expanded and functionally diverged angiosperm *CKX*s as non-ancient CKX groups.

Next, we focused on the comparison of ancient and non-ancient *CKX* genes in angiosperms in their expansion and expression patterns. The ancient *CKX* genes are generally conservatively retained in a single copy per species, with the exception of a few of these genes in angiosperms that have undergone recent polyploidization events, as observed in *G. max*. In contrast, most non-ancient *CKX* genes have undergone significant expansions. Our synteny analysis using MCScanX^30^ showed that 54 of 128 non-ancient *CKX* genes in angiosperms likely resulted from WGD/segmental duplication and 20 from tandem or proximal duplication (Table S7). Based on our analysis, most of the WGD/segmental *CKX* duplicates may have more likely been derived from WGD rather than segmental duplication because (1) all the WGD/segmental duplicates except for *ClCKX2/7* come from angiosperms that have experienced lineage-specific polyploidization events; (2) the pairwise Ks values of the WGD/segmental duplicates are in agreement with the ages of these polyploidization events (Table S7); and (3) the vast majority of the randomly selected collinear regions containing WGD/segmental duplicates are involved in the WGD remnants reported in previous studies (data not shown). Therefore, WGD duplication is likely a major mechanism of the expansion of non-ancient *CKX* genes in angiosperms.

To investigate the expression patterns of the *CKX* genes, we summarized the data reported previously in *Arabidopsis*^31^, maize^32^, and woodland strawberry^33^. The results demonstrate that in tissues/organs, ancient *CKX* genes (i.e., *ZmCKX10*) are nearly constitutively expressed, whereas non-ancient *CKX* genes exhibit divergent expression patterns (Fig. S5a, c, e). The non-ancient *CKX* genes have also diversified in response to abiotic stresses (Fig. S5b, d, f). At least one member of the duplicate *CKX*s in each non-ancient *CKX* group responded positively to abiotic stress treatment. For instance, within the duplicate pair *AtCKX1*/*6* in the angiosperm-derived Group VI, the expression level of *AtCKX6* was significantly increased under drought, cold or salinity stress conditions (Fig. S5b). In contrast, the ancient *CKX*s, such as *AtCKX7* and *ZmCKX10*, showed no significant response to abiotic stresses (Fig. S5b, d). These results suggest that in angiosperms, the ancient *CKX*s likely maintain constitutive and non-abiotic stress-responsive expression patterns throughout the plant, while the non-ancient *CKX*s showing functional divergence after duplication confer tissue/organ-specific and abiotic stimulus-responsive expression patterns.

### Expression patterns of *CKX* genes upon dehydration

**Fig. 4:**
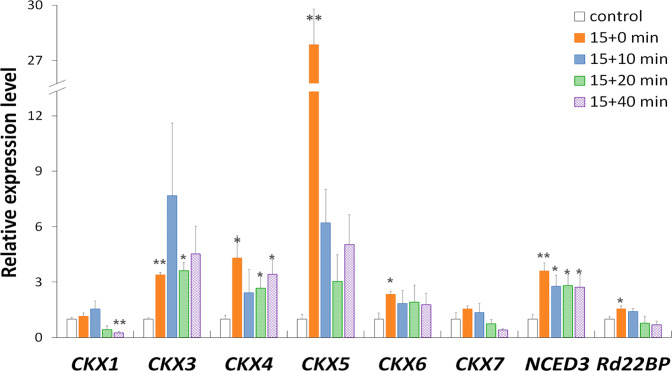
Rapid responses of representative *Arabidopsis CKX* genes in leaves under dehydration. Leaves from three-week-old *Arabidopsis* seedlings were exposed to fanning for accelerated dehydration for approximately 15 min to achieve 10% water loss, followed with 0 (15+0), 10 (15+10), 20 (15+20) or 40 (15+40) min of incubation in sealed plastic bags. Detached leaves placed under 100% relative humidity without treatment were used as controls, in which expression levels were set to 1. The *Tub8* gene was used as an endogenous reference gene. The error bar indicates the standard error of the mean (SEM) (*n* = 3 biological replicates), and an asterisk indicates a significant difference between treated leaves and the control (* for *p* < 0.05, ** for *p* < 0.01).

To verify the differences in abiotic stress responses between the ancient and non-ancient *CKX* genes, we examined the expression profile of *AtCKX* genes in the very early stages of dehydration treatment. *Arabidopsis* leaves were air-dried for 15 min, resulting in approximately 10% water loss. At the end of the 15 min dehydration period, significant increases in the expression of two drought-induced marker genes, *NCED3* and *Rd22BP1*, were observed (Fig. 4), indicating that the dehydration treatment was effective. Among the six expressed *AtCKX* genes, four of the five non-ancient *CKX*s, *AtCKX3-6*, showed significantly increased expression levels (Fig. 4). This increase in expression declined 10–20 min after the initiation of dehydration, but the expression levels of *AtCKX3-6* throughout the duration of the stress treatment were consistently higher than those under the control condition. The rapid upregulation of most non-ancient *AtCKX* genes at very early stages of dehydration strongly suggests that non-ancient *CKX*s play an important role in the plant response to water stress. On the other hand, the ancient *CKX AtCKX7* did not present significant increases in expression under dehydration, indicating that the ancient *CKX* genes are less involved in the regulation of abiotic stress responses in angiosperms.

### Non-ancient *CKX* genes play a role in the oxidative and drought stress tolerance of angiosperms

**Fig. 5:**
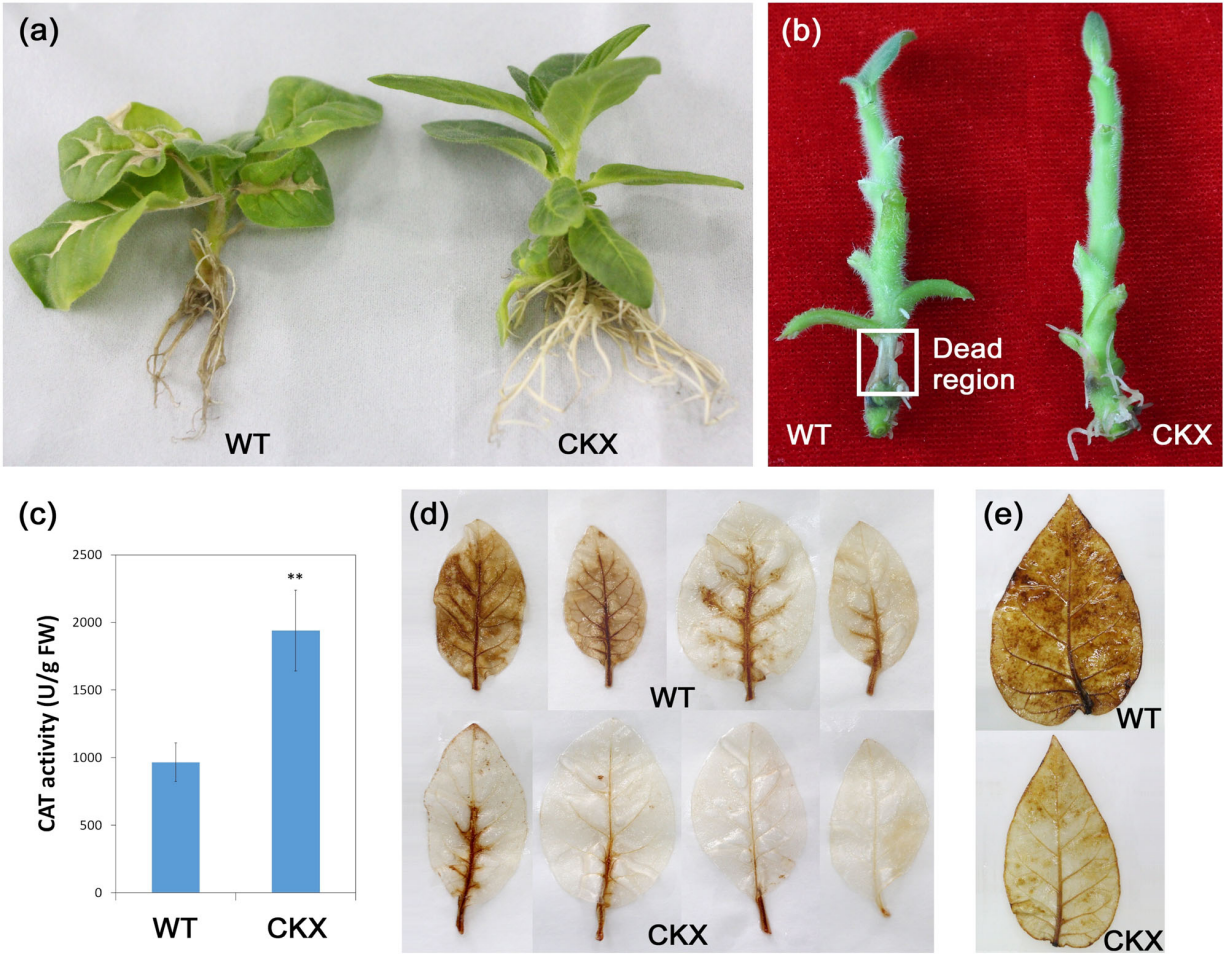
Improved oxidative stress tolerance in transgenic tobacco plants overexpressing a non-ancient *CKX* gene. **a**,**b** More dead tissues were observed in WT (left) than *35S:AtCKX2*-overexpressing (CKX2-10, right) tobacco plants under oxidative stress for 5 days. **c** Lower catalase (CAT) enzyme activities were observed in the leaves of WT plants than in the leaves of CKX2-10 plants under oxidative stress for 36 h. The error bars indicate SEM values (*n* = 3 biological replicates), and asterisks indicate significant differences between WT and transgenic plants (** for *p* < 0.01). **d**, **e** More H_2_O_2_ accumulation was observed in WT leaves (upper) than in CKX2-10 leaves (lower) under oxidative stress for 36 h (**d**) or under drought treatment (**e**; see Fig. S7) after 14 days.

We further investigated the role of the non-ancient *CKX* genes in plant tolerance to abiotic stresses. We generated transgenic tobacco plants overexpressing the non-ancient *CKX* gene *AtCKX2* under the control of the 35S promoter. The *CKX*-overexpressing plants displayed typical cytokinin-deficient phenotypes, including an expanded root system with increased root branching and a narrower leaf shape than the wild-type plants (Figs. 5a, S6). However, the height of the transgenic plants was similar to that of the wild-type plants when either cultured on MS medium or grown in the soil (Figs. 5a, S7).

Oxidative stress tolerance is a basis of tolerance to many abiotic stresses, such as drought and extreme temperatures. We examined the oxidative stress tolerance of the wild-type (WT) and *CKX* transgenic plants through the application of methyl viologen (MV), a chemical that can generate reactive oxygen species (ROS) in plant cells. Tissue death starting in the petioles followed by the veins of WT leaves and necrosis in WT stem tissues were observed after 2–3 days of culture in MV-containing media (Fig. 5a, b). In contrast, the transgenic plants remained healthy with no obvious symptoms under the same conditions. Significantly higher levels of H_2_O_2_ were detected in WT plant leaves than in their transgenic counterparts after 36 h of exposure to MV (Fig. 5d). We also observed an approximately 2-fold increase in ROS-scavenging activity (catalase enzymes) in the transgenic leaves (Fig. 5c). These results show that the overexpression of a non-ancient *CKX* gene can lead to increased ROS-scavenging activity in plant cells and increased plant tolerance to oxidative stress.

Increased tolerance was also observed in the *CKX*-overexpressing plants compared with the WT plants under drought conditions. Leaves were harvested from WT and *CKX* transgenic plants at two weeks after watering was ceased. Much less H_2_O_2_ accumulation was detected in transgenic leaves than in the WT controls (Fig. 5e), indicating that the *CKX*-overexpressing plants show higher tolerance to oxidative stress induced by drought. Moreover, although the wild-type and *CKX* transgenic plants displayed similar degrees of wilting and stem shrinking after one month of drought treatment (Fig. S7b, d), the transgenic plants showed significantly faster and better recovery than the WT plants (Fig. S7c, e). More than 90% of the 60 transgenic plants recovered from drought stress, as indicated by fully turgid leaves and stems, within 4 days after rewatering. In contrast, 75% of the 60 wild-type plants failed to recover, and the rest took 5 days or longer to reach full turgor. Therefore, the overexpression of a non-ancient *CKX* resulted in improved plant tolerance to drought, in addition to oxidative stress (Table 1).

**Table 1:** Comparison of stress-related functions between ancient and non-ancient *AtCKX* genes.

Phenotype		Ancient *AtCKX7*	Non-ancient *AtCKX*s	Reference
Rapid drought response^a^		No	Yes	This study
Stress tolerance^b^	Oxidative stress	Not reported	Increased	This study
	Drought	Not reported	Increased	This study,^12,18^
	High temperature	Not reported	Increased	^14,18^
	Salinity	Not reported	Increased	^12^
Root growth^b^	Primary roots	Reduced	Increased	This study,^11,13^
	Lateral roots	Reduced	Increased	This study,^11,13^
Protein property	Subcellular localization^c^	Cytosol	ER, vacuole or apoplast	^11,13,40^
	Substrate preference^d^	*c*Zs	iPs and/or *t*Zs	^2,28^

^a^Rapid drought response refers to the response to drought treatment within 15 min

^b^Results of *CKX* overexpression under the control of the 35S promoter are shown

^c^The reduction of cytokinins by cytosolic AtCKX7 resulted in the early termination of primary root growth^13^, whereas reduction by other AtCKXs resulted in increased primary and lateral root growth and development (Fig. S6)^11,38^

^d^Under stress conditions, the levels of *t*Zs were reduced, but the levels of *c*Zs were increased^12,41^, suggesting a negative role of *t*Zs, but not *c*Zs, in the stress response

## Discussion

### Origin of land plant *CKX* genes

Our analyses show that, with the sole exception of the excavate *N. gruberi*, *CKX* homologs are restricted to land plants and bacteria. Although the existence of putative *CKX* homologs has been suggested in several other eukaryotes, such as a few microalgal species^34^, no *CKX* homologs could be identified in the complete genomes of these species. Hence, the ability to degrade cytokinins in these other eukaryotes may not involve CKX proteins. The distribution of *CKX* homologs mainly in land plants and bacteria suggests that HGT has likely occurred during the evolution of this gene, though other possible scenarios (particularly differential gene losses) cannot be completely excluded. Furthermore, the identification of *CKX*s in multiple bacterial phyla indicates that *CKX* genes may have evolved in ancient bacteria and subsequently spread to land plants.

A well-known route of gene transfer from bacteria to plants is endosymbiotic gene transfer (EGT) from mitochondria and plastids^21^. Schmülling et al.^20^ once hypothesized a plastid origin of plant *CKX* genes due to the presence of *CKX* homologs in cyanobacteria. Nevertheless, the phylogenies constructed here and previously by others^4^ do not support that hypothesis. Instead, our analyses indicate that land plant *CKX*s are most closely related to chlamydial sequences, suggesting a chlamydial origin of these genes. Chlamydiae are a group of obligate intracellular bacteria living in animals, free-living amoebae and the environment^25^. Chlamydial genes in photosynthetic eukaryotes have been reported from several genome analyses^22–25^. Because of the nature of Chlamydiae as obligate intracellular endosymbionts, it has been suggested that an ancient chlamydial endosymbiont was present in the most recent common ancestor of Plantae. Thus, genes were transferred from this chlamydial endosymbiont to the nucleus of the host cell, which in turn facilitated the establishment of cyanobacterial endosymbionts (i.e., plastids). We conclude that the original/ancient *CKX* in land plants was most likely derived from a gene in the ancient chlamydial endosymbiont and that its expansion and diversification produced the non-ancient *CKX*s, which might contribute more to the regulation of organ development and abiotic stress responses in angiosperms.

### Evolution of *CKX* genes in angiosperms

It is unclear from the available data when the *CKX* gene was transferred from the chlamydial endosymbiont to the nuclear genome of plants and coopted into their cytokinin metabolism. Nevertheless, neither a CKX homolog nor CKX activity has been detected in any algal lineage, suggesting that cytokinin degradation by CKXs in Plantae is most likely restricted to land plants. Our analyses indicate that the single ancient *CKX* gene underwent duplications in the common ancestor of seed plants, after which further duplications and divergence occurred in angiosperms, giving rise to non-ancient *CKX*s with different features from the ancient genes (Fig. 3b). The percentage of *CKX* genes among the total genes in most sampled angiosperms was nearly stable (Table S6). More notably, in the vast majority of sampled angiosperms, the ratio of *CKX* to *IPT* genes (the *IPT* genes encode key enzymes responsible for the biosynthesis of cytokinins) is restricted to a value of approximately 1 (Table S8). This result indicates that the numbers of *CKX* and *IPT* genes in angiosperm genomes may be kept in balance and vary concomitantly. As such, the retention of *CKX* and *IPT* duplicates in angiosperms likely follows the gene balance hypothesis, which postulates that complex subunits or proteins in a regulatory cascade need to be maintained in dosage balance to avoid negative consequences^35^. CKX proteins exhibit opposite roles to IPTs in the control of endogenous cytokinin content. Thus, the dosage balance between *CKX* and *IPT* genes may be critical for the regulation of endogenous cytokinins in angiosperms.

### Functional divergence of *CKX* genes in angiosperms

There are two types of *IPT* genes, *tRNA-IPT*s (ancient cytokinin biosynthesis genes) and *ATP/ADP*-*IPT*s (non-ancient cytokinin biosynthesis genes), in angiosperms. In an accompanying article on this topic (Wang et al.^36^), we show that in angiosperms, the *tRNA-IPT* (ancient) genes are conservatively retained, constitutively expressed throughout the plant, and nonresponsive or only slightly responsive to environmental stresses. On the other hand, the expression of *ATP/ADP-IPT*s (non-ancient), which emerged and diverged in angiosperms, is tissue specific and is rapidly downregulated under various stress conditions. It is particularly interesting that we found similar characteristics between the ancient/non-ancient *CKX* genes and the ancient/non-ancient *IPT* (*tRNA-*type and *ATP/ADP*-type) genes in terms of their evolution, tissue/organ expression and environmental stress responses in angiosperms (Table 2). Based on several lines of evidence discussed below, we hypothesize that, similar to the *ATP/ADP*-*IPT* (non-ancient) genes (Wang et al.^36^), the non-ancient *CKX*s play regulatory roles in organ development and the adaption of angiosperms to environmental stresses, whereas the ancient *CKX*s more likely exhibit housekeeping functions, similar to the *tRNA-IPT* (ancient) genes.

**Table 2:** Evolutionary history, protein characteristics and proposed roles of cytokinin metabolism genes in angiosperms.

	*tRNA-IPT*s	*ATP/ADP-IPT*s	Ancient *CKX*s	Non-ancient *CKX*s
Products or substrates^a^	*c*Zs	iPs/*t*Zs	Preferentially *c*Zs	Preferentially iPs/*t*Zs
Origin	Ancient *IPT* in the last common ancestor of eukaryotes	Class II *tRNA-IPT* in the common ancestor of angiosperms	Chlamydial *CKX* during the primary endosymbiosis	Ancient *CKX* in the last common ancestor of land plants
Expansion & functional divergence in angiosperms	No	Yes	No	Yes
Organ development of angiosperms	Mutations lead to small and often chlorotic plants^5^	Altered expression leads to plants with abnormal organ development^5^	Overexpression leads to no significant changes after root growth is restored two weeks after germination^13,b^	Altered expression leads to plants with abnormal organ development^37,61^
Responses to abiotic stresses	Slightly responsive or nonresponsive	Downregulated	Nonresponsive	Rapidly induced
Abiotic stress tolerance	Not reported	Knockout mutations lead to increased drought or salt tolerance^12^; stress-, maturation-, or senescence-induced overexpression of the *Agrobacterium AMP-IPT* leads to increased iPs/*t*Zs and improved tolerance to drought, salt and heat stresses^12,62^	Not reported	Overexpression leads to improved tolerance to drought, salt^62^, heat^14^, or oxidative stresses, and knockout mutations of an inflorescence meristem-specific *CKX* lead to improved salt tolerance^63^
Proposed roles in angiosperms	Housekeeping	Regulatory	Housekeeping	Regulatory

^a^Products of IPTs or substrates of CKXs

^b^Plants overexpressing the ancient *AtCKX7* gene showed small shoots and early-terminating primary roots within two weeks after germination. Thereafter, root growth was restored, and no significant changes were observed in plant growth or developmental patterns at later stages^13^

First, the differences in copy numbers and expression patterns of the ancient and non-ancient *CKX* genes support the notion that these two types of *CKX* genes (ancient and non-ancient) functionally diversified in angiosperms. Our results show that the ancient *CKX* genes are conservatively retained in 1 or 2 copies in most sampled angiosperms, whereas the non-ancient *CKX* genes have undergone significant expansions and display large variations in gene numbers (from 3 to 14) among different species. More importantly, based on the analyses of the data reported previously in *Arabidopsis*^31^, maize^32^, and woodland strawberry^33^, the ancient *CKX* genes are nearly constitutively expressed across all tissues/stages, while the non-ancient *CKX*s exhibit highly diversified expression patterns (Fig. S5). In particular, the expression levels of most non-ancient *CKX*s vary significantly among different tissues/organs or developmental stages. The relatively constitutive expression of the ancient *CKK* genes suggests that these genes likely play a housekeeping role in maintaining basic cellular functions, while the highly diversified expression patterns of the non-ancient *CKX*s suggest regulatory roles in plant growth and development.

Second, plants with altered expression of ancient and non-ancient *CKX*s exhibit distinct phenotypic changes. With respect to organ development, the knockout of non-ancient *CKX*s leads to increased floral and ovule primordium formation and more flowers, siliques, and seeds but abnormal floral organ development, based on the observation of *Arabidopsis ckx3 ckx5* double mutants^37^. Constitutive overexpression of single non-ancient *CKX*s improves primary root growth and lateral root formation while substantially reducing shoot development, decreasing the activity of vegetative and floral shoot apical meristems and leaf primordia, and causing lower fertility^11^. In contrast, young seedlings constitutively overexpressing the ancient *AtCKX7* gene produce normal primary roots immediately after germination, but their growth ceases three days later^13^. The root growth of the *AtCKX7*-overexpressing seedlings is restored after one week. The aerial parts of the *AtCKX7*-overexpressing plants are relatively small before their root growth is restored. However, there are no significant changes observed in plant growth and developmental patterns at later stages after root growth is restored^13^. Furthermore, under tissue culture conditions, no developmental changes except for reduced root sizes have been observed in the isolated roots of transgenic tomato plants that overexpress an ancient *CKX* gene, *ZmCKX10*^29^. These results also support the housekeeping and regulatory roles of ancient and non-ancient *CKX*s, respectively, in the organ development of angiosperms.

Third, the ancient and non-ancient *CKX* genes exhibit differential response patterns upon environmental stress treatments (Table 1). According to our analyses of *CKX* expression data in this and previous studies^31–33^, the ancient copies appear to be constitutively expressed in tissues/organs and to show no responses to abiotic stresses (Figs. 4, S5). In contrast, the non-ancient *CKX*s display rapid and diversified responses to stresses. Among the species containing lineage-specific duplicates in the ancient *CKX* group (e.g., woodland strawberry), one member (*FveCKX6*) displays nearly the same pattern as the singleton ancient *CKX* genes *AtCKX7* and *ZmCKX10*, while the other member (*FveCKX7*) shows diversified expression in tissues/organs and positive responses to abiotic stresses, similar to the members of the non-ancient *CKX* groups. These findings suggest that the ancient *CKX* genes tend to be retained with a conserved copy number and play a housekeeping role, while the non-ancient *CKX*s are functionally diversified and more involved in the adaptation of angiosperms to environmental stresses.

Fourth, the manipulation of the expression of ancient and non-ancient *CKXs* likely leads to different changes in stress tolerance (Table 1). This and previous studies have demonstrated that the overexpression of a non-ancient *CKX* gene improves plant tolerance to drought, osmotic, or heat stress (Fig. S7)^12,18,38^. Moreover, our experiments revealed that transgenic plants overexpressing a non-ancient *CKX* gene exhibit increased oxidative stress tolerance (Fig. 5). Lower H_2_O_2_ accumulation under drought (Fig. 5e) and increased antioxidant defense against abiotic stresses have also been detected. Environmental stresses usually lead to an increase in ROS generation, which triggers oxidative stress in plants^39^. Increased oxidative stress tolerance provides a basis for the improvement of tolerance to other stresses. Furthermore, the improved root growth and biomass (Fig. S6)^11,38^ of the plants overexpressing a non-ancient *CKX* play a role in the improvement of overall drought tolerance. On the other hand, the suppression of root growth following the overexpression of an ancient *CKX* suggests that ancient *CKX*s may not play a positive role in the environmental stress tolerance of angiosperms.

In addition, ancient and non-ancient CKXs present differential subcellular localizations (Table 1). Studies on AtCKXs have revealed that the ancient AtCKX7 uniquely localizes to the cytosol, whereas the non-ancient AtCKXs predominantly localize to the endoplasmic reticulum (ER), vacuole or apoplast^11,13,40^. This discrepancy in subcellular localizations between ancient and non-ancient CKXs is likely related to their respective housekeeping and regulatory roles in angiosperms. Taken together, these above lines of evidence support our hypothesis that the non-ancient *CKX* genes are more involved in the regulation of organ development and the adaptation of angiosperms to environmental stresses, whereas the ancient *CKX*s likely contribute more to maintaining basic cellular functions.

The ancient and non-ancient CKXs also exhibit different substrate specificities as well. Non-ancient CKXs, such as AtCKX4 and ZmCKX1, present a greater substrate preference for *t*Z- or iP-type cytokinins, while ancient CKXs prefer *c*Zs^11,28,29^. Experiments in *CKX*-overexpressing plants also show that the non-ancient AtCKXs degrade *t*Zs much more efficiently than the ancient AtCKX7, whereas AtCKX7 preferentially degrades *c*Zs^13^. We have proposed the hypothesis in the accompanying article in this issue (Wang et al.^36^) that iPs/*t*Zs play more regulatory roles in organ development and stress responses, while *c*Zs play a housekeeping role in maintaining basic cellular functions, based on the analyses of the corresponding genes responsible for their biosynthesis (*ATP/ADP-IPT*s and *tRNA-IPT*s). In this study, we provide further supporting evidence for this hypothesis from comparative analyses of non-ancient and ancient *CKX*s (Table 2). We have demonstrated above that the non-ancient CKXs, preferentially degrading iPs/*t*Zs, play a regulatory role, while the ancient CKXs, preferentially degrading *c*Zs, play a housekeeping role in the organ development of angiosperms. We have also shown that non-ancient *CKX*s are rapidly induced, whereas ancient *CKX*s are not responsive under environmental stress conditions. These results are consistent with the observations that the levels of iPs/*t*Zs are drastically reduced, while those of *c*Zs are either not changed or increased in response to environmental stresses^41^. Moreover, plants overexpressing non-ancient *AtCKX*s show substantially reduced levels of iP/*t*Z-cytokinins and exhibited strong drought- or salt stress-tolerant phenotypes, regardless of their *c*Z levels^12^. These findings support the notion that iPs/*t*Zs, rather than *c*Zs, play a regulatory role in abiotic stress response/tolerance in angiosperms. In addition, due to increases in *c*Z concentrations under stress conditions, it has been suggested that *c*Zs may play a role in maintaining basic cellular functions under growth-limiting conditions^2,41^. However, because *c*Zs have been shown to present much less cytokinin activity than iPs and *t*Zs^42,43^, the role of *c*Zs under stress conditions needs to be experimentally verified.

In conclusion, based on the results from the accompanying (Wang et al.^36^) and the current studies, we propose that in angiosperms, the ancient *tRNA-IPTs* and *CKX*s and their products or preferred substrates, the *c*Z-type cytokinins, play a housekeeping role to maintain basic cellular functions. On the other hand, the non-ancient *ATP/ADP-IPTs* and *CKXs* and their products or preferred substrates, the iP- and *t*Z-type cytokinins, contribute more to the regulation of organ development and abiotic stress responses. Our results and hypotheses shed light on the differential roles of the ancient and non-ancient *IPT* and *CKX* genes and associated *c*Z-, iP-/*t*Z-type cytokinins in plant growth and development and the responses to abiotic stresses. We hope that our hypothesis may bring about more interest regarding the elucidation of the functions of both types of cytokinins as well as the genes involved in their biosynthesis and metabolism in angiosperms.

## Material and methods

### Data retrieval

An exhaustive search for *CKX* genes was performed in online GenBank databases (http://blast.ncbi.nlm.nih.gov/) and our customized local databases. All available complete proteomes and corresponding genomic sequences from land plants, bacteria, and other organisms were downloaded from the NCBI (ftp://ftp.ncbi.nlm.nih.gov/) and JGI databases (http://genome.jgi.doe.gov/). Additional searches of *CKX* genes in green and red algae, glaucophytes and seedless land plants were performed online in the database of the One Thousand Plants Consortium (1KP; https://www.bioinfodata.org/Blast4OneKP/). To estimate the percentages of presence of *IPT* and *LOG* genes, 44 complete genomes from representative lineages of bacteria, archaea, and eukaryotes as well as another six species from the major lineages of land plants, were sampled according to recently proposed classification systems (Table S2)^44,45^. The transcriptomic data used for the expression analyses of *FveCKX* genes in the tissues and organs of woodland strawberry (*Fragaria vesca* L.) were downloaded from the SGR database (http://bioinformatics.towson.edu/strawberry/)^33^.

### Homolog identification

The full-alignment Hidden Markov Model (HMM) profiles of the Cytokin-bind domains (Pfam: PF09265), IPPT domains (Pfam: PF01715), and Lysine_decarbox domains (Pfam: PF03641) of the CKX, IPT, and LOG proteins, respectively, were downloaded from the Pfam database v28.0^46^. These HMM profiles were used as queries to search for homologs in all available proteome sequences of bacteria, archaea, and eukaryotes using hmmsearch^47^ (E-value cut-off 10^−4^). Additional searches of the CKXs were performed in the online GenBank protein databases of archaea and eukaryotes other than land plants using BLASTP. Next, the sequences of all matching proteins were verified using the Pfam database (http://pfam.xfam.org/)^46^ and the Simple Modular Architecture Research Tool database (SMART; http://smart.embl-heidelberg.de/)^48^ with an E-value cut-off of 10^−10^. The identified protein sequences that contained the core domains of all known CKXs [both Cytokin-bind (Pfam: PF09265) and FAD_binding_4 (Pfam: PF01565) domains], IPTs [single IPPT (Pfam: PF01715) domain], and LOGs [single Lysine_decarbox (Pfam: PF03641) domain] were regarded as putative homologs in the study.

For homolog searches of CKX proteins against genomic or other nucleotide sequences, the full-alignment HMM profile of the Cytokin-bind domain was used as a query in TBLASTN searches with an E-value cut-off of 10^−4^. To include all possible functional homologs, hit sequences longer than 10% of the domain (30 amino acids) were selected for further verification. The nucleotide hit sequences together with their 5′ and 3′ flanking regions (each 5000 bp) were used for gene annotation by FGENESH (http://linux1.softberry.com/berry.phtml). The genes containing the hit sequences or genes that were reannotated using FGENESH were considered as putative candidates. The domain structure of their protein sequences was assessed using the Pfam and SMART databases. The proteins containing both Cytokin-bind and FAD_binding_4 domains were regarded as CKX homologs.

### Sequence alignment and phylogenetic analysis

For phylogenetic analyses of *CKX* genes in eukaryotes and bacteria, six representative land plants, one excavate, and 49 bacteria from all major bacterial groups that contained CKX homologs were sampled (Table S1). The FAD_binding_4 and Cytokin-bind domain regions of the CKX proteins were aligned using ClustalX 2.1^49^, followed by manual inspection and refinement. Gaps and ambiguously aligned sites were removed manually. The ClustalX parameters included a gap opening penalty of 5 and a gap extension penalty of 3. The WAG+G+F substitution model was identified as the optimal model of amino acid sequence evolution using the program MODELGENERATOR^50^ with four gamma categories. The phylogenetic trees were then constructed via Bayesian^51^ and maximum-likelihood (ML)^52^ analyses. The Bayesian analysis was performed with MrBayes 3.2^51^, where WAG was selected as the evolutionary model, and the number of discrete categories used to approximate the gamma distribution was set to 4 (rates = gamma, ngammacat = 4). The ML analyses were conducted with PhyML node-by-node SH test^53^ and RaxML^54^ bootstrap replicates using the WAG + F + G model, and 100 bootstrap replicates were selected to calculate the bootstrap support for the ML trees.

To investigate the evolution of land plant CKXs, 21 species with available whole-genome sequences were sampled among all major lineages of land plants (Table S6). *CKX* genes were identified using the above methods, and the phylogeny was constructed using PhyML based on the multiple sequence alignment of Cytokin-bind domain sequences. A functional bacterial CKX (RfaCKX1) was used as an outgroup. The ModelGenerator program was also used to identify the best-fitting model.

### Ka/Ks evaluation and synteny analysis

Ks and Ka values were calculated using the program yn00 from the PAML package^55^. The CKX alignments between the six land plants and 49 bacteria sampled in the MrBayes phylogeny were used. Nucleotide sequences were forced to fit the amino acid CKX alignments using PAL2NAL49. To determine the mechanisms responsible for the non-ancient *CKX* genes in angiosperms, all against all BLASTP searches (E-value cut-off of 10^−10^, top five matches) were first performed in each proteome of the 16 angiosperms sampled in the phylogeny of land plant CKXs (Table S6). Syntenic blocks were detected, and the origins of all non-ancient *CKX* genes in angiosperms obtained from BLASTP searches were classified using McScanX^30^. The mechanisms of the non-ancient *CKX* duplications were then retrieved. The pairwise Ks values of proximal, tandem and WGD/segmental *CKX* duplicates were calculated using the CKX alignments of each of the 16 angiosperms with the yn00 program^55^.

### Plant materials, tobacco transformation and growth conditions

The *Arabidopsis* Col-0 plants used for the dehydration treatment were grown in soil at 20 °C (dark)–24 °C (light) under 120 µmol m^−2^ s^−1^ irradiance, 65% relative humidity and a 16-h light/8-h dark photoperiod in a growth chamber.

An *Arabidopsis* non-ancient *CKX* gene, *AtCKX2*, was used to examine *CKX* overexpression in tobacco plants (*Nicotiana tabacum* L. var. *xanthi*). For plasmid construction, the full-length open reading frame of the *AtCKX2* cDNA was cloned into the *Apa*1 and *Bam*H1 sites of the binary vector *p*Cambia2301 (Clontech) under the control of the cauliflower mosaic virus (CaMV) 35S promoter to yield an overexpression plasmid for non-ancient *CKX*s. The correct orientations were confirmed by restriction digestion analysis and sequencing. The tobacco leaf-disc explants were used for transformation by *Agrobacterium tumefaciens* strain LBA4404 carrying the binary vector *p*Cambia2301.

After rooting in Murashige and Skoog (MS) medium containing 50 mg L^−1^ kanamycin, the transgenic plants were verified and vegetatively propagated in MS medium. Twenty-six independent lines were obtained, displaying different levels of cytokinin-deficient phenotypes^56^. Two lines (CKX2-10 and CKX2-21) showing obvious typical cytokinin-deficient phenotypes were selected and propagated for further oxidative and drought stress experiments. Free-hand sections of the shoots of wild-type (WT), *35S*:*AtCKX2* and *Agrobacterium IPT*-overexpressing tobacco plants were cultured on MS medium^57^ in Magenta boxes at 22 °C (dark)–26 °C (light) under 120 µmol m^−2^ s^−1^ light and a 16-h light/8-h dark photoperiod in a growth chamber. Photographs of the roots of these plants were taken after four weeks of growth.

### Leaf dehydration treatment and qRT-PCR analysis

The rosette leaves of 3-week-old *Arabidopsis* Col-0 plants were harvested and placed between a piece of gauze and a piece of filter paper and air-dried using a fan for accelerated dehydration for approximately 15 min until 10% water loss was achieved. The leaves were then divided into four samples, which were immediately packed in foil and plunged into liquid nitrogen after 0, 10, 20, or 40 min of incubation. Detached leaves that were placed on wet filter paper under 100% relative humidity were used as controls.

Total RNA was extracted from the samples using the SV Total RNA Isolation System (Takara). cDNA was synthesized using the PrimeScript RT reagent Kit with a DNA Eraser kit (Perfect Real-Time) (Takara) according to the manufacturer’s instructions. qRT-PCR was performed in a Bio-Rad IQ5 Real-Time PCR System (Bio-Rad) using SYBR Premix *Ex* Taq (Takara). Three biological replicates were conducted, and the results for each sample were normalized using *Tub8* as an internal reference gene. Transcription levels are presented as 2^-ΔCt^ values, where ΔCt represents the difference between the cycle threshold values of the target and the reference genes^58^. The statistical significance of the differences between the treated leaves and the control was estimated using the one-tailed *t*-test. The primers used for the qRT-PCR analyses are provided in Table S9.

### Oxidative treatment using methyl viologen (MV)

Free-hand sections of the shoots of WT or transgenic plants were cultured for three weeks. Thereafter, one WT and one *35S*:*AtCKX2* tobacco plant were transferred to Murashige and Skoog medium^57^ containing 20 μM MV in each box. Fifteen CKX2-10, fifteen CKX2-21, and thirty WT plants were used for each experiment. The experiments were repeated three times, and all the experiments showed similar results. Leaves were harvested after 36 h for the detection of H_2_O_2_ accumulation and antioxidant enzyme activities with three biological replicates. Photographs of representative oxidatively stressed plants (WT and CKX2-10) were taken after 5 days of treatment.

### Drought stress treatment

After one month of growth in MS media, one WT and one *35S*:*AtCKX2* tobacco plant generated from the free-hand sections of the shoots were transferred to each pot. The plants were grown at 22 °C (dark)–26 °C (light) under 160 µmol m^−2^ s^−1^ irradiance, 65% relative humidity and a 16-h light/8-h dark photoperiod in a growth chamber and fertilized once a week with 300 mL of 1% solid fertilizer (20% N, 20% P, 20% K, 0.05% Mg and 0.025% Mn). After one month, watering was stopped, followed by one month of drought treatment. Ten CKX2-10, ten CKX2-21, and twenty WT plants were used for each experiment. The experiments were repeated three times, and all the experiments showed similar results. On Day 14, leaves with no sign of wilting were harvested for the detection of H_2_O_2_ accumulation with three biological replicates. Photographs of the drought-stressed plants (WT and CKX2-10) were taken on Day 31 before rewatering. Then, the plants were rewatered (the soil was saturated with water), and photographs were taken on the 3rd and 14th days after rewatering.

### DAB staining and enzyme assays

H_2_O_2_ production by the oxidatively or drought-stressed leaves was detected by 3,3′-diaminobenzidine (DAB) polymerization^59^. For catalase enzyme analyses, frozen oxidatively stressed leaf samples (0.2 g) were homogenized in 1.6 mL of sodium phosphate buffer (50 mM, pH 7.8). The homogenate was then centrifuged for 20 min at 12,000 × *g*, and the supernatant was used for the detection of enzyme activity according to Zhou et al.^60^.

## Supplementary information

Supplementary materials
