# Supplementary material for: Evolution and roles of cytokinin genes in angiosperms 2: Do ancient CKXs play housekeeping roles while non-ancient CKXs play regulatory roles?
Source: Hortic Res. 2020 Mar 1;7:29. doi: 10.1038/s41438-020-0246-z (PMC7049301; doi:10.1038/s41438-020-0246-z)
Supplement: Supplementary file 1 — Supplementary materials [file 41438_2020_246_MOESM1_ESM.docx]

**Supplementary information**

**Article title:** Evolution and roles of cytokinin genes in angiosperms 2: Do ancient CKXs play housekeeping roles while non-ancient CKXs play regulatory roles?

**Journal:** Horticulture Research

**Authors:** Xiaojing Wang, Jing Ding, Shanshan Lin, Decai Liu, Tingting Gu, Han Wu, Robert N. Trigiano, Richard McAvoy, Jinling Huang, Yi Li

**Corresponding authors:**

**Yi Li**, State Key Laboratory of Crop Genetics and Germplasm Enhancement and College of Horticulture, Nanjing Agricultural University, Nanjing, P. R. China; Department of Plant Science and Landscape Architecture, University of Connecticut, Storrs, CT 06269, USA; e-mail: yi.li@uconn.edu;

**Jinling Huang**, State Key Laboratory of Crop Stress Adaptation and Improvement, Key Laboratory of Plant Stress Biology, School of Life Sciences, Henan University, Kaifeng, China; Department of Biology, East Carolina University, Greenville, North Carolina 27858, USA; e-mail: huangj@ecu.edu;

**Jing Ding**, State Key Laboratory of Crop Genetics and Germplasm Enhancement and College of Horticulture, Nanjing Agricultural University, Nanjing, P. R. China; e-mail: jding@njau.edu.cn.

**
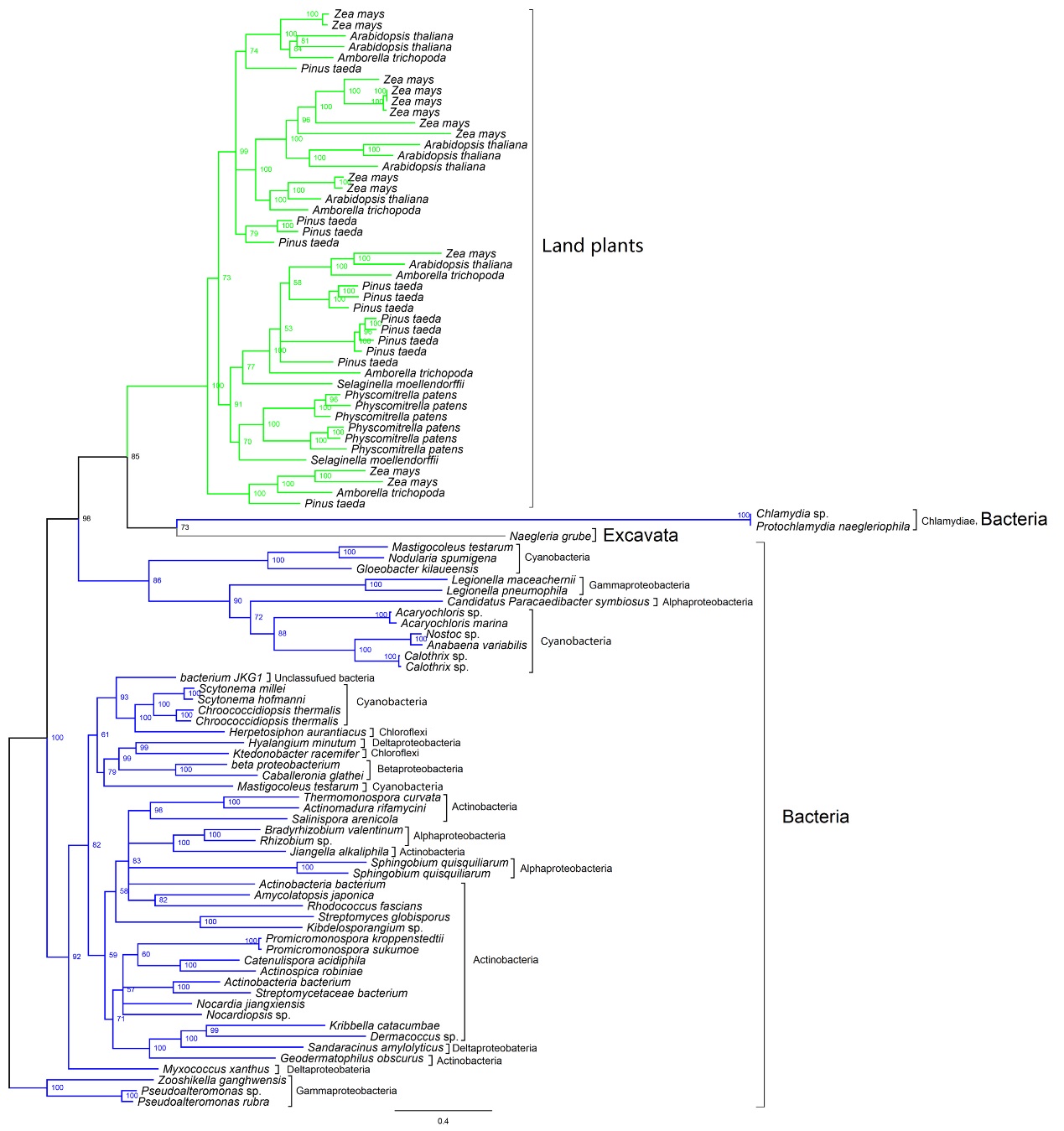
**

**Fig. S1. Phylogenetic analyses demonstrate a close relationship between land plant and chlamydial CKXs.** The phylogeny shows detailed MrBayes tree for Fig. 2, which was constructed based on the two-domain regions of the CKX proteins in the six representative land plants, one excavate, and 49 selected bacteria (see Table S1) using MrBayes 3.2. Support values (Bayesian posterior probabilities×100) greater than 50 are indicated at the nodes. Color coding: Green, land plants; blue, bacteria; gray, excavate.

**
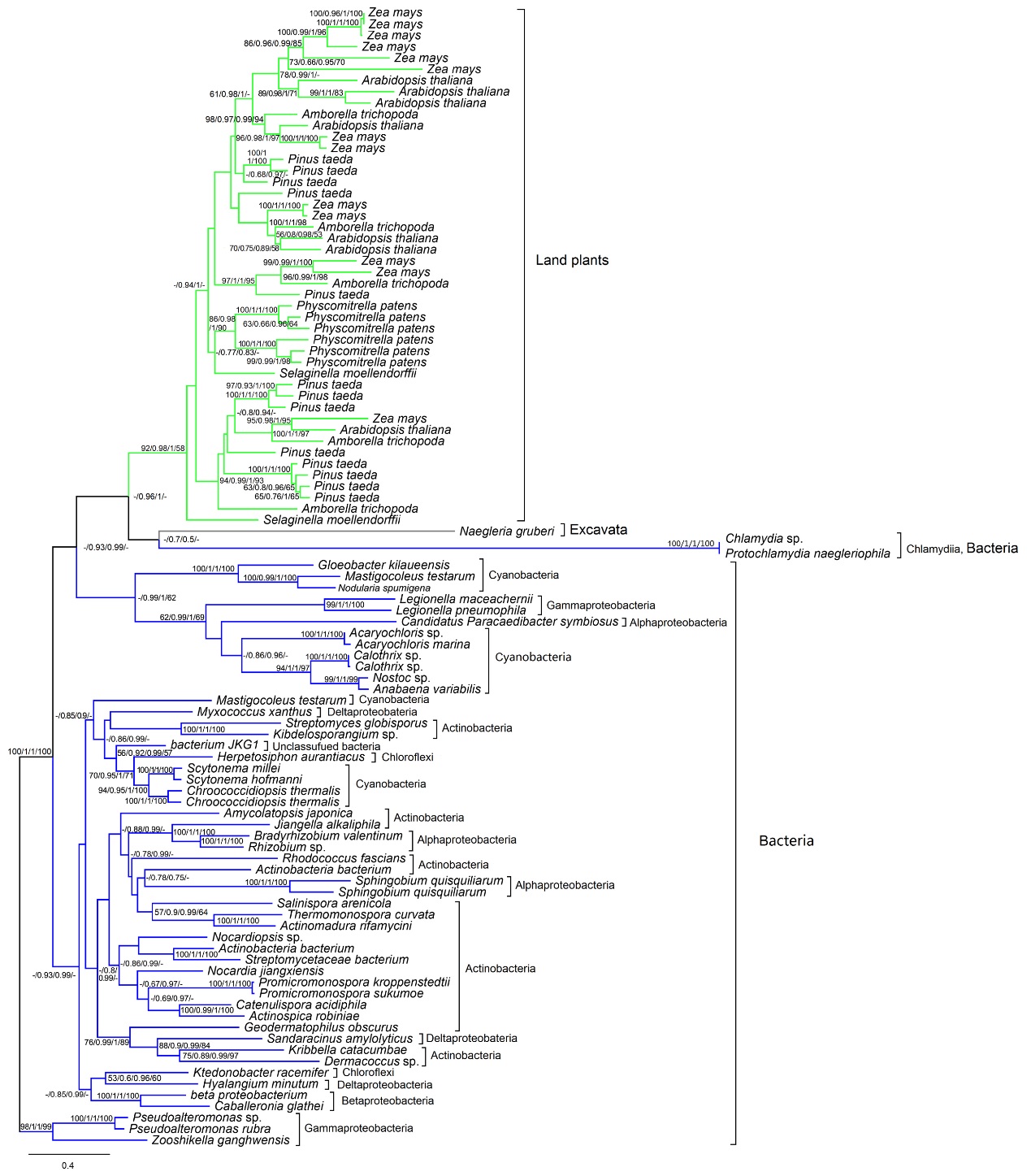
**

**Fig. S2. The PhyML phylogeny of CKX proteins in bacteria and eukaryotes supports the close relationship of land plant and chlamydial CKXs.** The tree was constructed based on the two-domain regions of the CKX proteins in the six representative land plants, one excavate, and 49 selected bacteria (see Table S1) using PhyML. The topology is confirmed by support values of the key nodes between plant and bacterial branches, which are indicated in the order PhyML bootstrap/Bayesian posterior probability/PhyML node-by-node SH test/RAxML bootstrap. “-” represents support values lower than 50. Color coding: Green, land plants; blue, bacteria; gray, excavate.


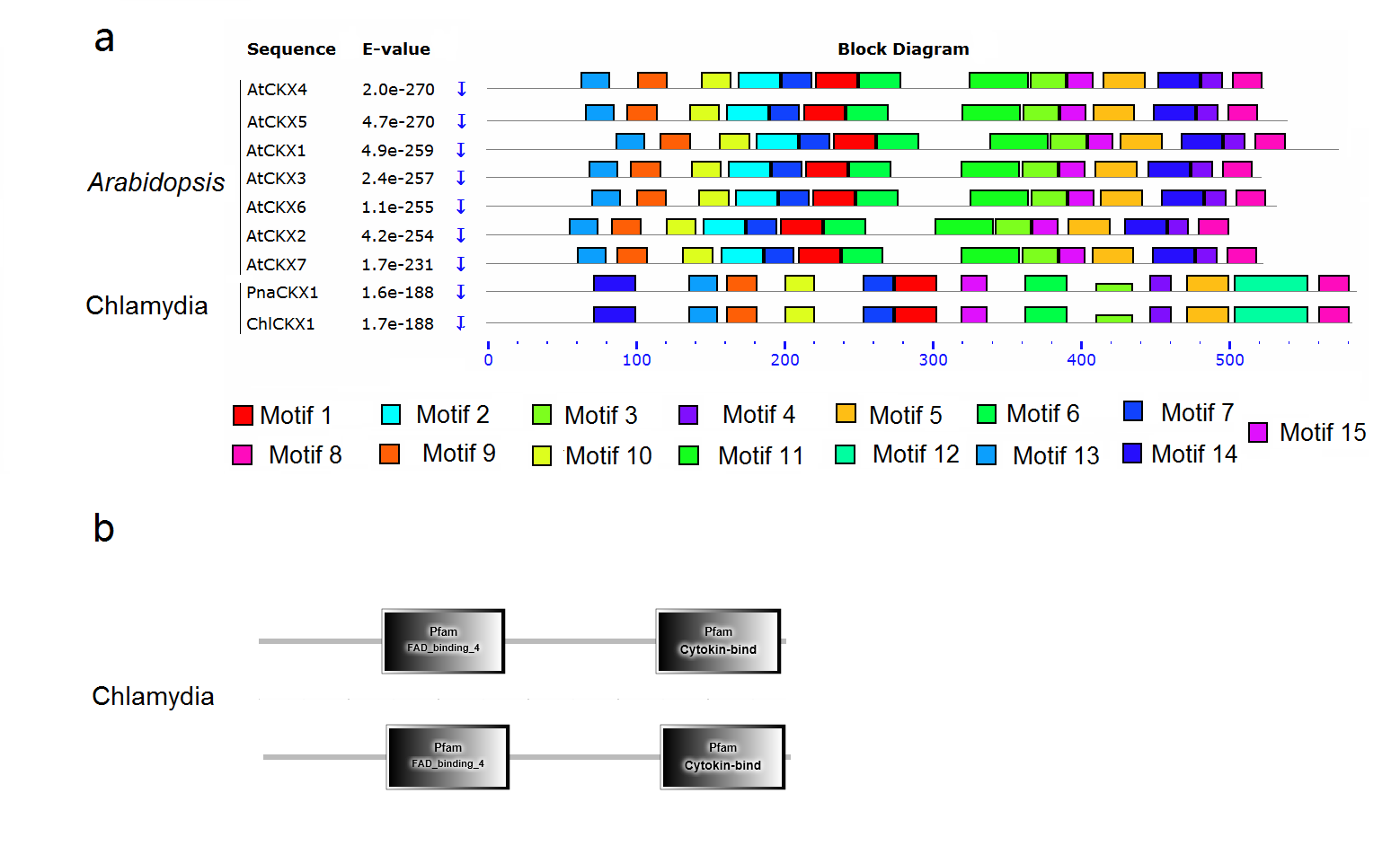


**Fig. S3.** **Schematic diagram of amino acid motifs (a) and core domains (b) of *Arabidopsis* and chlamydial CKXs.** **a** Motif analysis was performed using MEME5.0.1 as described in the methods. Fifteen motifs (1 to 15) were identified and indicated by different colors. Motif location and combined p-value are shown. **b** Domain analysis was performed using SMART as described in the methods.


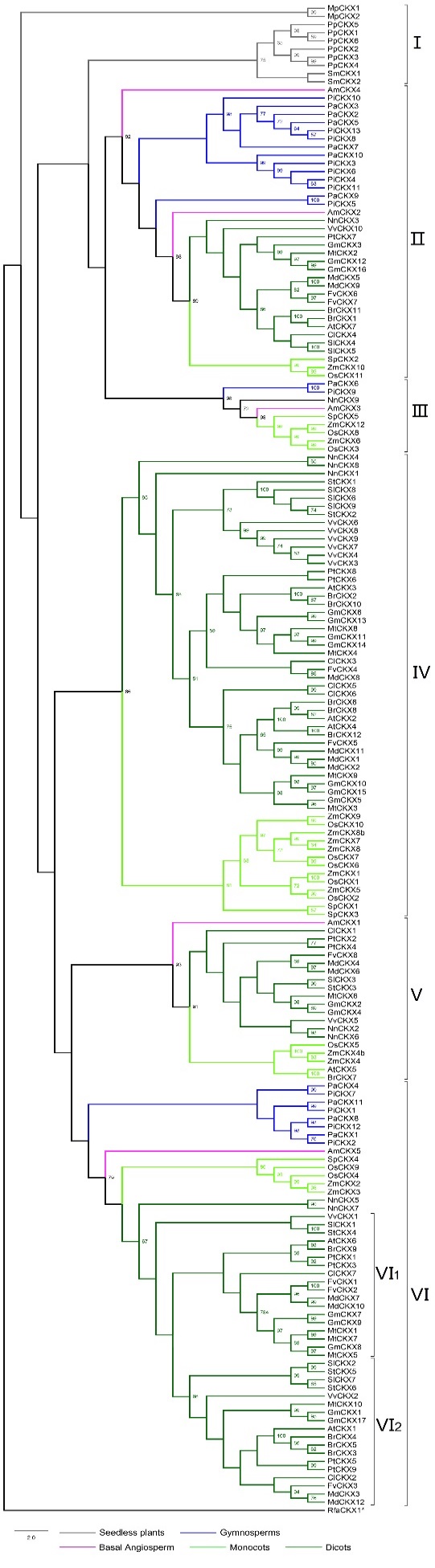


**Fig. S4. The Phylogeny of land plant CKXs from 21 representative species shows significant expansion of the gene family in angiosperms.** The tree was constructed using PhyML based on the cytokin-bind domain regions of the CKX proteins. Bootstrap values greater than 50 are shown. The color coding for the plant lineages is indicated at the bottom. The abbreviations for the species names in front of the gene names are indicated here and in Table S6: for seedless plants (grey branches): Mp: *Marchantia polymorpha*, Pp: *Physcomitrella patens*, Sm: *Selaginella moellendorffii*; for gymnosperms (blue branches): Pa: *Picea abies*, Pi: *Pinus taeda*; for the basal angiosperm (pink branches): Am: *Amborella trichopoda*; for monocots (light green branches): Os: *Oryza sativa*, Sp: *Spirodela polyrhiza*, Zm: *Zea mays*; for dicots (green branches): At: *Arabidopsis thaliana*, Br: *Brassica rapa*, Cl: *Citrus clementina*, Fv: *Fragaria vesca*, Gm: *Glycine max*, Md: *Malus x domestica*, Mt: *Medicago truncatula*, Nn: *Nelumbo nucifera*, Pt: *Populus trichocarpa*, Sl: *Solanum lycopersicum*, St: *Solanum tuberosum*, Vv: *Vitis vinifera*.

**
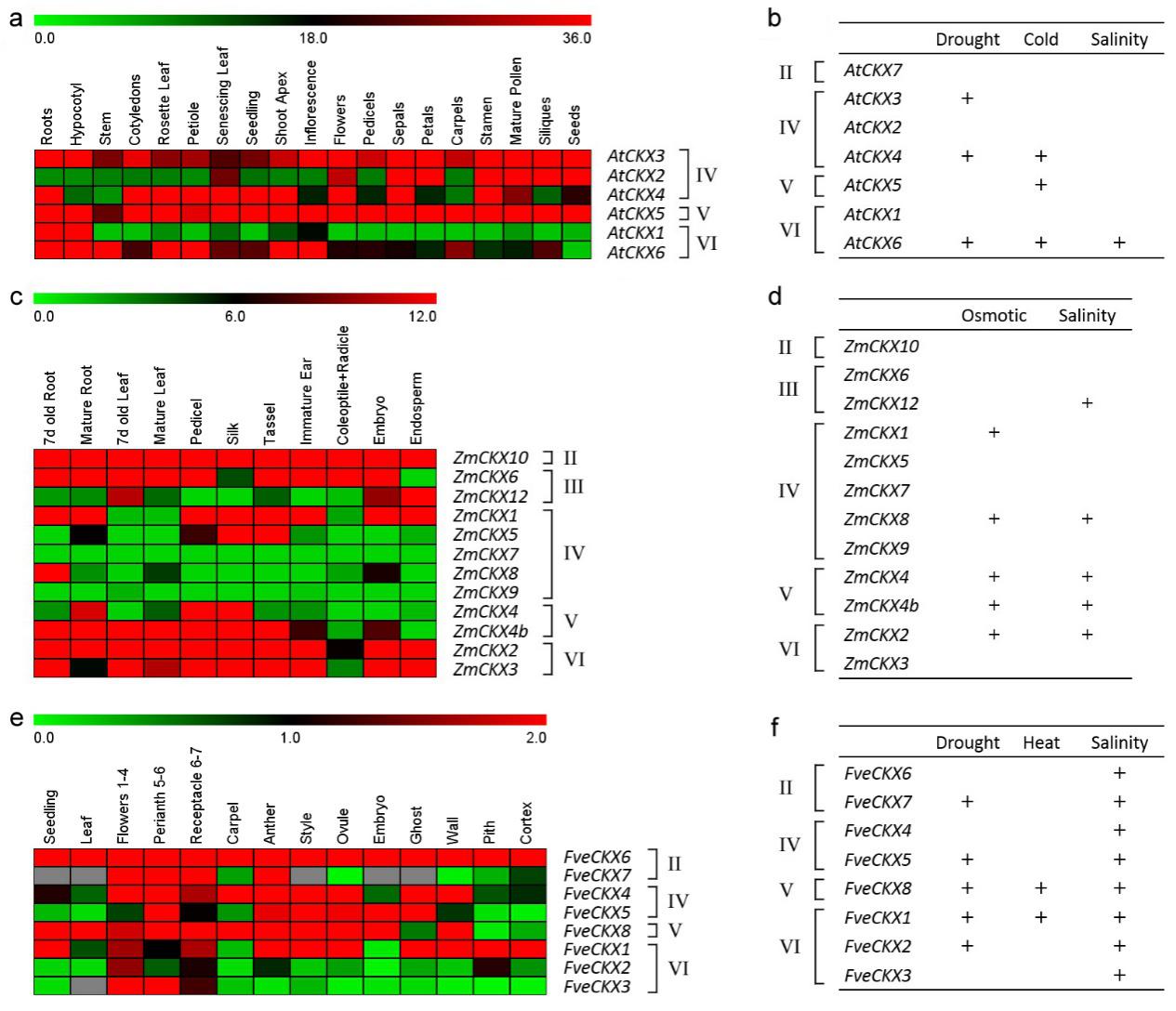
Fig. S5. Phylogeny and differential expression patterns of *Arabidopsis* (a, b), maize (c, d), and woodland strawberry (e, f) *CKX* genes. a, b** Tissue/organ (**a**) and stress-induced (**b**) expression patterns of *AtCKX* genes were summarized based on the Expression Atlas of Arabidopsis Development (AtGenExpress, data for *AtCKX7* is unavailable, http://bar.utoronto.ca/efp/cgi-bin/efpWeb.cgi) and tiling microarray data^1^, respectively. **c, d** Tissue/organ (**c**) and stress-induced (**d**) expression patterns of *ZmCKX* genes were summarized based on qPCR results^2^. **e, f** Tissue/organ (**e**) and stress-induced (**f**) expression patterns of *FveCKX* genes were summarized based on transcriptomic data from the SGR database ([http://bioinformatics.towson.edu/strawberry/](#_ENREF_1))^3^ and qPCR results^4^, respectively. The heat map shows log2 “relative RPKM values” of individual *FveCKX* genes. Gray boxes indicate undetectable expression of the genes. Numbers after flower tissues represent their developmental stages. The plus (+) indicates expression or significant increase of *CKX* expression levels (expression ratios (stressed/control) > 1.5).


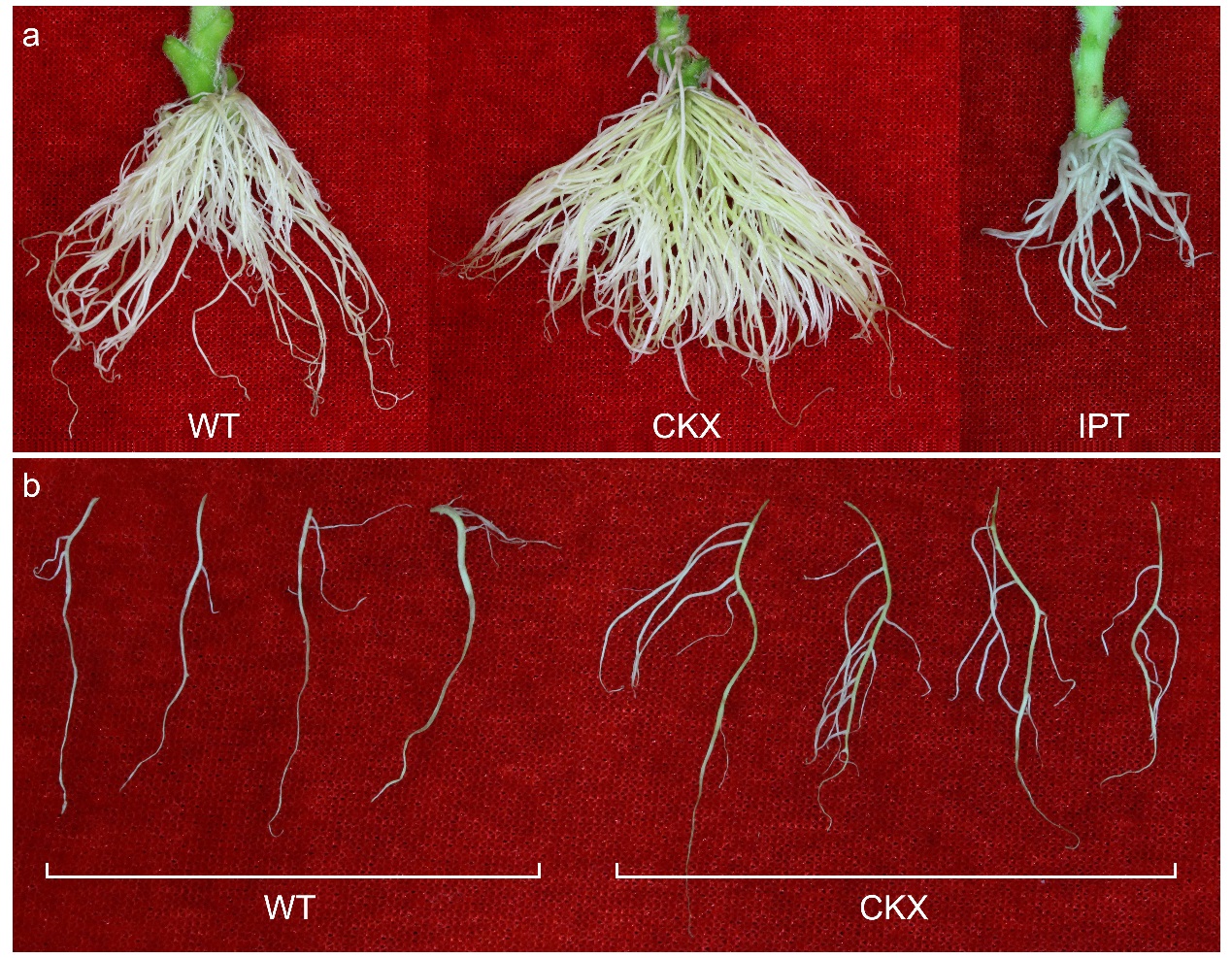


**Fig. S6. Increased root growth is observed in transgenic tobacco plants overexpressing a non-ancient *CKX*gene.** (**a**) Root systems of WT (left), *35S:AtCKX2* (CKX2-10, middle) and *IPT* (right) plants after four-week culture in a MS medium. (**b**) Enhanced lateral root formation and growth in CKX2-10 plants (right) compared with the WT (left).

**
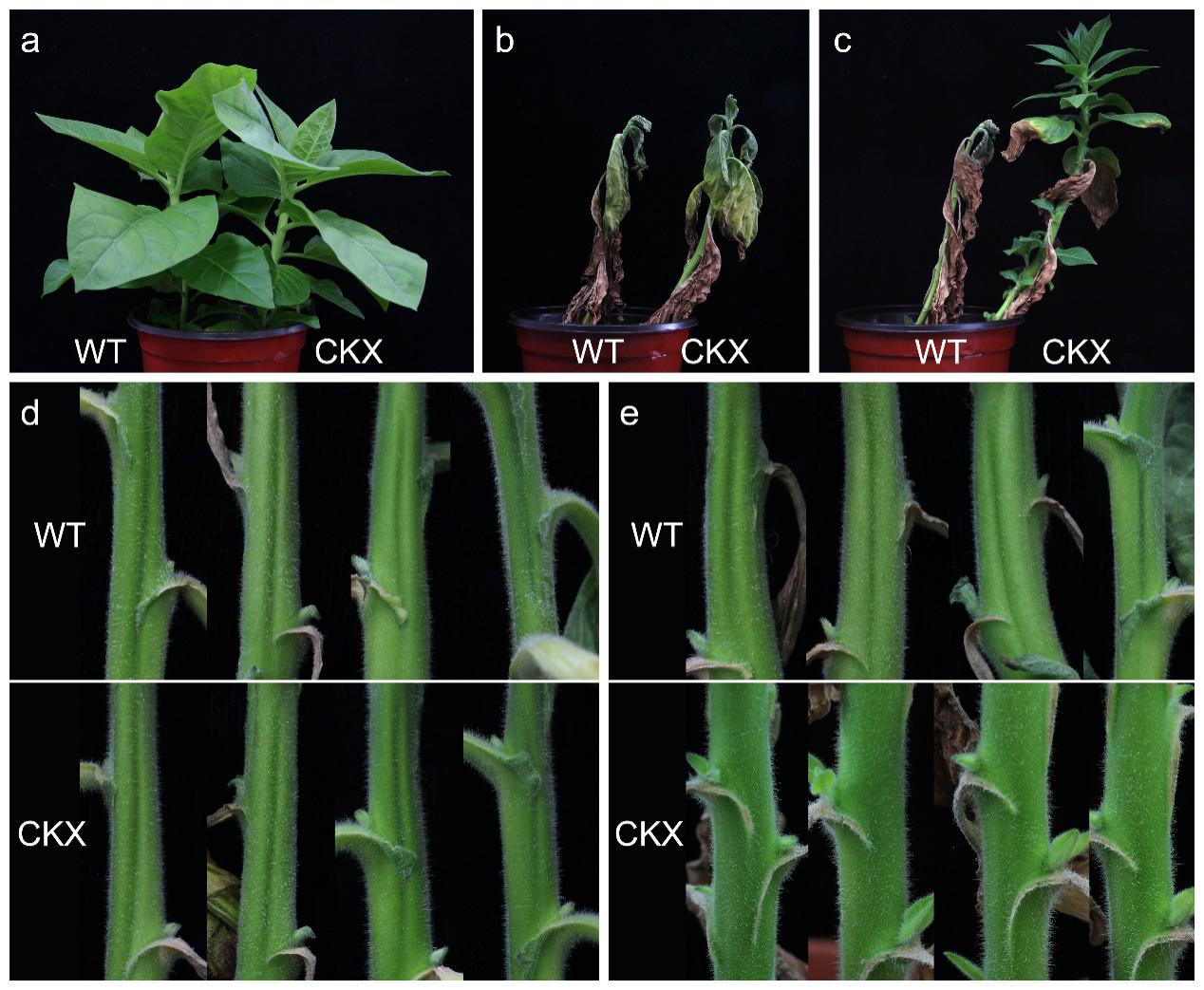
**

**Fig. S7. Improved drought tolerance is observed in transgenic tobacco plants overexpressing a non-ancient *CKX*gene.** (**a**-**c**) WT (left) and *35S:AtCKX2* overexpressing (CKX2-10, right) tobacco plants before drought treatment (**a**), one month after the stop of watering (**b**), and two weeks after re-watering (**c**) of the plants in (**b**). (**d**) Stem sections of WT (upper) and CKX2-10 (lower) plants show similar stress responses (stem shrinking) one month after the stop of watering. (**e**) The drought-stressed plants were then watered and 3 days after, the CKX2-10 stems fully recovered but WT stems did not as indicated with shrinking.

**Table S1.** List of the excavate and bacterial species sampled in the MrBayes tree in Figs. 2 and S1 and the PhyML tree in Fig. S2.

| Taxon | Phylum or class | Strain or species |
| --- | --- | --- |
| Excavate | Percolozoa | *Naegleria gruberi* |
| Bacteria | Actinobacteria | *Actinobacteria bacterium* OK074 |
|  |  | *Kribbella catacumbae* |
|  |  | *Jiangella alkaliphila* |
|  |  | *Actinospica robiniae* |
|  |  | *Catenulispora acidiphila* DSM 44928 |
|  |  | *Kibdelosporangium* sp. MJ126-NF4 |
|  |  | *Amycolatopsis japonica* |
|  |  | *Salinispora arenicola* CNS-205 |
|  |  | *Dermacoccus* sp. Ellin185 |
|  |  | *Promicromonospora kroppenstedtii* |
|  |  | *Promicromonospora sukumoe* |
|  |  | *Nocardiopsis* sp. CNS-639 |
|  |  | *Actinomadura rifamycini* |
|  |  | *Thermomonospora curvata* |
|  |  | *Streptomycetaceae bacterium* MP113-05 |
|  |  | *Streptomyces globisporus* |
|  |  | *Geodermatophilus obscurus* |
|  |  | *[Nocardia jiangxiensis](https://www.ncbi.nlm.nih.gov/genome/14480?genome_assembly_id=42225)* [NBRC 101359](https://www.ncbi.nlm.nih.gov/genome/14480?genome_assembly_id=42225) |
|  |  | *Rhodococcus fascians* D188 |
|  | Chloroflexi | *Herpetosiphon aurantiacus* DSM 785 |
|  |  | *Ktedonobacter racemifer* DSM 44963 |
|  | Cyanobacteria | *Acaryochloris marina* MBIC11017 |
|  |  | *Acaryochloris* sp. CCMEE 5410 |
|  |  | *Chroococcidiopsis thermalis* PCC 7203 |
|  |  | *Scytonema millei* VB511283 |
|  |  | *Mastigocoleus testarum* |
|  |  | *Calothrix* sp. PCC 7507 |
|  |  | *Nodularia spumigena* |
|  |  | *Nostoc* sp. PCC 7120 |
|  |  | *Anabaena variabilis* ATCC 29413 |
|  |  | *Scytonema hofmanni* UTEX 2349 |
|  |  | *Gloeobacter kilaueensis* JS1 |
|  | Alphaproteobacteria | *Sphingobium quisquiliarum* P25 |
|  |  | *Bradyrhizobium valentinum* |
|  |  | *Rhizobium* sp. IRBG74 |
|  |  | *Candidatus Paracaedibacter symbiosus* |
|  | [Betaproteobacteria](https://www.ncbi.nlm.nih.gov/Taxonomy/Browser/wwwtax.cgi?mode=Tree&id=28216&session=1z5mW3seKtCJw-s_GSgK5LlzyfsPgN4NV8fBejyFcjP_8e5cuad5vwnnjnqpcW6ul3sOCc8dwydQ04IftkH3Ocm0_a08o4ugB8Zp6EAslhVp8dnJqzbLbEyaYweprpxWPpcMsef&opsess=1TcLj4b8VpiOIZyhJp7pg8P-m_Vo5a6dkc5e7_7160XRo6G_0PrB6Ns_jh-QLhqooQuL8jgxbns6njPu8ecPy-ljruyQjTbBuMf7xZrhiljdeGZNHMMYb03GmYAdCMllu4Enomc&en=1128421) | *Beta proteobacterium* AAP51 |
|  |  | *Caballeronia glathei* |
|  | Deltaproteobacteria | *Sandaracinus amylolyticus* |
|  |  | *Hyalangium minutum* |
|  |  | *Myxococcus xanthus* DK 1622 |
|  | Gammaproteobacteria | *Zooshikella ganghwensis* |
|  |  | *Pseudoalteromonas* sp. SM9913 |
|  |  | *Pseudoalteromonas rubra* |
|  |  | *Legionella maceachernii* |
|  |  | *Legionella pneumophila subsp. Pneumophila* str. Philadelphia 1 |
|  | Chlamydiae | *Protochlamydia naegleriophila* |
|  |  | *Chlamydia* sp. 'Diamant' |
|  | Unclassified bacteria | Bacterium JKG1 |

**Table S2.** A survey of *IPT*, *LOG* and *CKX* homologs in the sampled living organisms indicates a wide distribution of *IPTs* and *LOGs* but a sporadic distribution of *CKXs* in bacteria and eukaryotes other than land plants.

| **Domain** | **Supergroup** | **Phylum or group** | **Species** | **IPT** | **LOG** | **CKX** |
| --- | --- | --- | --- | --- | --- | --- |
| **Bacteria** |  | Parcubacteria | *Parcubacteria group* bacterium GW2011_GWA2_37_10 | 1 | 0 | 0 |
|  |  | Berkelbacteria | *Candidatus Berkelbacteria* bacterium CG2_30_43_20 | 1 | 0 | 0 |
|  |  | Microgenomates | *Candidatus Amesbacteria* bacterium W2011_GWA2_42_12 | 0 | 1 | 0 |
|  |  | Cyanobacteria | *Nostoc* sp. PCC 7120 | 1 | 1 | 1 |
|  |  | Firmicutes | *Alicyclobacillus acidocaldarius* LAA1 | 1 | 0 | 0 |
|  |  | Chloroflexi | *Anaerolineae* bacterium CG2_30_58_95 | 0 | 1 | 0 |
|  |  | Atribacteria | *Candidatus Atribacteria* bacterium CG2_30_33_13 | 1 | 0 | 0 |
|  |  | Ignavibacteria | *Ignavibacteria* bacterium CG1_02_37_35 | 2 | 1 | 0 |
|  |  | Elusimicrobia | *Elusimicrobia* bacterium GWA2_61_42 | 1 | 1 | 0 |
|  |  | Rokubacteria | *Candidatus Rokubacteria* bacterium 13_1_40CM_4_69_39 | 1 | 1 | 0 |
|  |  | Spirochaetes | *Leptonema illini* DSM 21528 | 1 | 2 | 0 |
|  |  | Alphaproteobacteria | *Rhizobium* sp. YS-1r | 1 | 2 | 1 |
|  |  | Zetaproteobacteria | *Mariprofundus ferrooxydans* M34 | 1 | 2 | 0 |
|  |  | Acidithiobacillia | *Thermithiobacillus tepidarius* DSM 3134 | 1 | 2 | 0 |
|  |  | Betaproteobacteria | *Beta proteobacterium* AAP51 | 1 | 1 | 1 |
|  |  | Gammaproteobacteria | *Oceanimonas* sp. GK1 | 1 | 0 | 0 |
| **Archaea** |  | Micrarchaeota | *Candidatus Micrarchaeota archaeon* RBG_16_36_9 | 0 | 0 | 0 |
|  |  | DPANN | *Archaeon* GW2011_AR21 | 0 | 0 | 0 |
|  |  | Nanohaloarchaeota | *Candidatus Haloredivivus* sp. G17 | 0 | 0 | 0 |
|  |  | Altiarchaeales | *Candidatus Altiarchaeales archaeon* IMC4 | 0 | 0 | 0 |
|  |  | Theionarchaea | *Theionarchaea archaeon* DG-70 | 0 | 0 | 0 |
|  |  | Methanobacteria | *Methanobacterium paludis* strain SWAN1 | 0 | 0 | 0 |
|  |  | Crenarchaeota | *Thermogladius cellulolyticus* 1633 | 0 | 0 | 0 |
|  |  | Lokiarchaeaota | *Lokiarchaeum* sp. GC14_75 | 0 | 2 | 0 |
| **Eukarya** | SAR | Stramenopiles | *Thalassiosira pseudonana* | 1 | 1 | 0 |
|  |  | Alveolata | *Tetrahymena thermophila* | 1 | 0 | 0 |
|  |  | Cercozoa | *Plasmodiophora brassicae* | 2 | 0 | 0 |
|  |  | Foraminifera | *Reticulomyxa filosa* | 1 | 0 | 0 |
|  | Excavata | Euglenozoa | *Trypanosoma cruzi* Dm28c | 1 | 1 | 0 |
|  |  | Metamonada | *Spironucleus salmonicida* | 0 | 0 | 0 |
|  | Amoebozoa | Dictyostelia | *Dictyostelium discoideum* AX4 | 3 | 1 | 0 |
|  |  | Discosea | *Acanthamoeba castellanii* str. Neff | 2 | 1 | 0 |
|  | Opisthokonta | Fungi | *Saccharomyces cerevisiae* | 1 | 1 | 0 |
|  |  | Fonticula | *Fonticula alba* | 2 | 1 | 0 |
|  |  | Ichthyosporea | *Sphaeroforma arctica* JP610 | 2 | 0 | 0 |
|  |  | Choanomonada | *Salpingoeca rosetta* | 2 | 0 | 0 |
|  |  | Metazoa | *Homo sapiens* | 1 | 0 | 0 |
|  | Incertae sedis | Haptophyta | *Chrysochromulina* sp. CCMP291 | 1 | 1 | 0 |
|  |  | Cryptophyta | *Chroomonas mesostigmatica* | 1 | 2 | 0 |
|  |  | Apusomonadida | *Thecamonas trahens* ATCC 50062 | 2 | 1 | 0 |
|  | Archaeplastida | Glaucophyta | *Cyanophora paradoxa* | 1 | 2 | 0 |
|  |  | Rhodophyta | *Cyanidioschyzon merolae* strain 10D | 1 | 2 | 0 |
|  |  | Chlorophyta | *Volvox carteri* | 1 | 1 | 0 |
|  |  | Charophyta | *Klebsormidium nitens* | 1 | 2 | 0 |
|  |  | Embryophyta | *Physcomitrella patens* | 6 | 10 | 6 |
|  |  |  | *Selaginella moellendorffii* | 1 | 3 | 2 |
|  |  |  | *Pinus taeda L.* | 3 | 16 | 14 |
|  |  |  | *Amborella trichopoda* | 5 | 8 | 5 |
|  |  |  | *Zea mays* | 11 | 13 | 13 |
|  |  |  | *Arabidopsis thaliana* | 9 | 10 | 7 |

**Table S3.** List of sequenced land plants used for the survey of *CKX* genes.

| Species | Species | Species | Species | Species |
| --- | --- | --- | --- | --- |
| *Actinidia chinensis* | *Capsicum annuum* | *Hevea brasiliensis* | *Oryza meridionalis* | *Setaria italica* |
| *Aegilops tauschii* | *Carica papaya* | *Hordeum vulgare* | *Oryza nivara* | *Setaria viridis* |
| *Amaranthus hypochondriacus* | *Chenopodium quinoa* | *Kalanchoe fedtschenkoi* | *Oryza punctata* | *Solanum lycopersicum* |
| *Amborella trichopoda* | *Cicer arietinum* | *Kalanchoe laxiflora* | *Oryza rufipogon* | *Solanum pennellii* |
| *Anacardium occidentale* | *Citrullus lanatus* | *Jatropha curcas* | *Oryza sativa* | *Solanum tuberosum* |
| *Ananas comosus* | *Citrus clementina* | *Lactuca sativa* | *Petunia axillaris* | *Sorghum bicolor* |
| *Aquilegia coerulea* | *Citrus sinensis* | *Linum usitatissimum* | *Phalaenopsis equestris* | *Sphagnum fallax* |
| *Arachis duranensis* | *Cleome hassleriana* | *Lotus japonicus* | *Phaseolus vulgaris* | *Sphagnum magellanicum* |
| *Arachis ipaensis* | *Cucumis melo* | *Lupinus angustifolius* | *Phoenix dactylifera* | *Spinacia oleracea* |
| *Arabidopsis halleri* | *Cucumis sativus* | *Malus domestica* | *Physcomitrella patens* | *Spirodela polyrhiza* |
| *Arabidopsis lyrata* | *Daucus carota* | *Manihot esculenta* | *Picea abies* | *Tarenaya hassleriana* |
| *Arabidopsis thaliana* | *Dichanthelium oligosanthes* | *Marchantia polymorpha* | *Pinus taeda* | *Theobroma cacao* |
| *Arabis alpina* | *Elaeis guineensis* | *Medicago truncatula* | *Panicum hallii* | *Trifolium pratense* |
| *Beta vulgaris* | *Erythranthe guttata* | *Mimulus guttatus* | *Panicum virgatum* | *Triticum aestivum* |
| *Boechera stricta* | *Eucalyptus grandis* | *Morus notabilis* | *Populus deltoides* | *Triticum urartu* |
| *Brachypodium distachyon* | *Eutrema salsugineum* | *Musa acuminata* | *Populus euphratica* | *Vigna angularis* |
| *Brachypodium stacei* | *Fragaria vesca* | *Nelumbo nucifera* | *Populus trichocarpa* | *Vigna radiata* |
| *Brachypodium sylvaticum* | *Genlisea aurea* | *Nicotiana benthamiana* | *Prunus avium* | *Vigna unguiculata* |
| *Brassica napus* | *Ginkgo biloba* | *Nicotiana sylvestris* | *Prunus mume* | *Vitis vinifera* |
| *Brassica oleracea* | *Glycine max* | *Nicotiana tomentosiformis* | *Prunus persica* | *Zea mays* |
| *Brassica rapa* | *Glycine soja* | *Olea europaea* | *Pyrus x bretschneideri* | *Ziziphus jujuba* |
| *Cajanus cajan* | *Gnetum montanum* | *Oropetium thomaeum* | *Ricinus communis* | *Zostera marina* |
| *Camelina sativa* | *Gossypium hirsutum* | *Oryza brachyantha* | *Salix purpurea* |  |
| *Capsella grandiflora* | *Gossypium raimondii* | *Oryza glaberrima* | *Selaginella moellendorffii* |  |
| *Capsella rubella* | *Helianthus vulgare* | *Oryza glumaepatula* | *Sesamum indicum* |  |

**Table S4.** A survey of *CKX* homologs in seedless plants in the 1KP database supports the ubiquitous presence of *CKX* genes in all major lineages of land plants.

| Lineage | No. of  all species | No. of species  with a *CKX* | Species with no *CKX* detected | Available nucleotides |
| --- | --- | --- | --- | --- |
| Liveworts | 26 | 21 | *Blasia* sp. | 3,003,945 |
|  |  |  | *Sphaerocarpos texanus* | 3,109,900 |
|  |  |  | *Ptilidium pulcherrimum* | 4,415,327 |
|  |  |  | *Schistochila* sp. | 5,182,815 |
|  |  |  | *Pellia* sp. (*cf epiphylla* (L*.*) *Corda*) | 4,649,166 |
| Mosses | 41 | 41 | / | / |
| Hornworts | 7 | 5 | *Leiosporoceros dussii* | 204,987 |
|  |  |  | *Anthoceros formosae* | 430,900 |
| Lycophytes | 21 | 21 | / | / |
| Eusporangiate monilophytes | 10 | 10 | / | / |
| Leptosporangiate moniophytes | 62 | 60 | *Osmundastrum cinnamomeum* | 3,002 |
|  |  |  | *Osmunda regalis* | 6,819 |
| Total | 167 | 158 | / | / |

**Table S5.** Ka/Ks analysis supports the closest relationship between land plant and chlamydial *CKX*s.

| Bacteria | Land plants | | |
| --- | --- | --- | --- |
|  | Average Ka | Average Ks | Average Ka/Ks |
| Chlamydiae | 1.213 | 2.784 | 0.436 |
| Cyanobacteria | 0.852*** | 3.145** | 0.274*** |
| Actinobacteria | 0.660*** | 3.586*** | 0.184*** |
| Chloroflexi | 0.677*** | 3.112 | 0.218** |
| Alphaproteobacteria | 0.791*** | 3.348* | 0.238*** |
| [Betaproteobacteria](https://www.ncbi.nlm.nih.gov/Taxonomy/Browser/wwwtax.cgi?mode=Tree&id=28216&session=1z5mW3seKtCJw-s_GSgK5LlzyfsPgN4NV8fBejyFcjP_8e5cuad5vwnnjnqpcW6ul3sOCc8dwydQ04IftkH3Ocm0_a08o4ugB8Zp6EAslhVp8dnJqzbLbEyaYweprpxWPpcMsef&opsess=1TcLj4b8VpiOIZyhJp7pg8P-m_Vo5a6dkc5e7_7160XRo6G_0PrB6Ns_jh-QLhqooQuL8jgxbns6njPu8ecPy-ljruyQjTbBuMf7xZrhiljdeGZNHMMYb03GmYAdCMllu4Enomc&en=1128421) | 0.649** | 3.610* | 0.180** |
| Deltaproteobacteria | 0.670** | 3.588*** | 0.187*** |
| Gammaproteobacteria | 0.911** | 3.188* | 0.287** |
| Unclassified bacteria^a^ | 0.660 | 3.638 | 0.181 |

Asterisks indicate significant difference between the chlamydial and other bacterial values using *t*-test (*, P < 0.05; **, P < 0.01; ***, P < 0.001).

^a^Significances of difference between the values of chlamydiae and unclassified bacteria could not be evaluated as the *CKX* was identified in only one bacterium from unclassified bacteria.

**Table S6.** The distribution of the land plant *CKX* genes among every group/subgroup of the phylogeny in Figs. 3 and S4 indicates *CKX* gene expansions.

| Group | Family | Species | Access | Abbrev | I | II | III | IV | V | VI | | Total | Percentage |
| --- | --- | --- | --- | --- | --- | --- | --- | --- | --- | --- | --- | --- | --- |
|  |  |  |  |  |  |  |  |  |  | VI_1_ | VI_2_ |  | (%) |
| Core eudicots | Brassicaceae | *Arabidopsis thaliana*^a^ | JGI | At | 0 | 1 | 0 | 3 | 1 | 1 | 1 | 7 | 0.026 |
|  | Brassicaceae | *Brassica rapa*^a^ | JGI | Br | 0 | 2 | 0 | 5 | 1 | 1 | 3 | 12 | 0.030 |
|  | Rutaceae | *Citrus clementina* | JGI | Cl | 0 | 1 | 0 | 3 | 1 | 1 | 1 | 7 | 0.020 |
|  | Salicaceae | *Populus trichocarpa*^a^ | JGI | Pt | 0 | 1 | 0 | 2 | 2 | 2 | 2 | 9 | 0.022 |
|  | Leguminosae | *Medicago truncatula*^a^ | JGI | Mt | 0 | 1 | 0 | 4 | 1 | 3 | 1 | 10 | 0.020 |
|  | Leguminosae | *Glycine max*^a^ | JGI | Gm | 0 | 3 | 0 | 7 | 2 | 3 | 2 | 17 | 0.030 |
|  | Rosaceae | *Fragaria vesca* | JGI | Fv | 0 | 2 | 0 | 2 | 1 | 2 | 1 | 8 | 0.024 |
|  | Rosaceae | *Malus x domestica*^a^ | JGI | Md | 0 | 2 | 0 | 4 | 2 | 2 | 2 | 12 | 0.019 |
|  | Vitaceae | *Vitis vinifera* | JGI | Vv | 0 | 1 | 0 | 6 | 1 | 1 | 1 | 10 | 0.040 |
|  | Solanaceae | *Solanum lycopersicum*^a^ | JGI | Sl | 0 | 2 | 0 | 3 | 1 | 1 | 2 | 9 | 0.026 |
|  | Solanaceae | *Solanum tuberosum*^a^ | JGI | St | 0 | 0 | 0 | 2 | 1 | 1 | 2 | 6 | 0.015 |
| Basal eudicot | Nelumbonaceae | *Nelumbo nucifera*^a^ | NCBI | Nn | 0 | 1 | 1 | 3 | 2 | 2 | | 9 | 0.034 |
| Monocots | Gramineae | *Zea mays*^a^ | JGI | Zm | 0 | 1 | 2 | 6 | 2 | 2 | | 13 | 0.020 |
|  | Gramineae | *Oryza sativa*^a^ | JGI | Os | 0 | 1 | 2 | 5 | 1 | 2 | | 11 | 0.028 |
|  | Lemnaceae | *Spirodela polyrhiza* | NCBI | Sp | 0 | 1 | 1 | 2 | 0 | 1 | | 5 | 0.025 |
| Basal angiosperm | Amborellaceae | *Amborella trichopoda* | JGI | Am | 0 | 2 | 1 | 0 | 1 | 1 | | 5 | 0.019 |
| Gymnosperms | Pinaceae | *Picea abies* | NCBI | Pa | 0 | 6 | 1 | 0 | 0 | 4 | | 11 | 0.017 |
|  | Pinaceae | *Pinus taeda* | NCBI | Pi | 0 | 8 | 1 | 0 | 0 | 4 | | 13 | 0.026 |
| Seedless plants | Selaginellaceae | *Selaginella moellendorffii* | JGI | Sm | 2 | 0 | 0 | 0 | 0 | 0 | | 2 | 0.009 |
|  | Funariaceae | *Physcomitrella patens* | JGI | Pp | 6 | 0 | 0 | 0 | 0 | 0 | | 6 | 0.023 |
|  | Marchantiaceae | *Marchantia polymorpha* | NCBI | Mp | 2 | 0 | 0 | 0 | 0 | 0 | | 2 | 0.010 |

^a^The monocot or eudicot species that has undergone lineage-specific polyploidization event(s).

**Table S7.** The tandem, proximal and WGD *CKX* duplicate pairs in angiosperms identified by McScanX indicate that WGD is a major mechanism of *CKX* expansion.

| Gene1 | Gene2 | Clade | Mechanism | Ks |
| --- | --- | --- | --- | --- |
| *MtCKX4* | *MtCKX3* | IV | Proximal | 0.3590 |
| *SlCKX8* | *SlCKX9* | IV | Proximal | 0.2763 |
| *VvCKX7* | *VvCKX9* | IV | Proximal | 0.0321 |
| *VvCKX9* | *VvCKX8* | IV | Proximal | 0.2145 |
| *SlCKX4* | *SlCKX5* | II | Tandem | 0.3810 |
| *ClCKX5* | *ClCKX6* | IV | Tandem | 0.1224 |
| *ClCKX3* | *ClCKX6* | IV | Tandem | 1.1778 |
| *ClCKX3* | *ClCKX5* | IV | Tandem | 0.3338 |
| *FvCKX4* | *FvCKX5* | IV | Tandem | 1.4095 |
| *GmCKX11* | *GmCKX10* | IV | Tandem | 1.2667 |
| *GmCKX15* | *GmCKX14* | IV | Tandem | 0.3365 |
| *MtCKX8* | *MtCKX9* | IV | Tandem | 0.7808 |
| *StCKX1* | *StCKX2* | IV | Tandem | 0.3827 |
| *VvCKX7* | *VvCKX8* | IV | Tandem | 0.2261 |
| *BrCKX1* | *BrCKX11* | II | WGD | 0.3082 |
| *GmCKX12* | *GmCKX16* | II | WGD | 0.2553 |
| *GmCKX3* | *GmCKX12* | II | WGD | 0.8755 |
| *GmCKX3* | *GmCKX16* | II | WGD | 0.8911 |
| *MdCKX5* | *MdCKX9* | II | WGD | 0.2411 |
| *OsCKX3* | *OsCKX8* | III | WGD | 1.3029 |
| *AtCKX2* | *AtCKX4* | IV | WGD | 1.4822 |
| *AtCKX2* | *AtCKX3* | IV | WGD | 0.8050 |
| *AtCKX3* | *AtCKX4* | IV | WGD | 1.4169 |
| *BrCKX10* | *BrCKX2* | IV | WGD | 0.2717 |
| *BrCKX2* | *BrCKX12* | IV | WGD | 1.5020 |
| *BrCKX6* | *BrCKX12* | IV | WGD | 0.7860 |
| *BrCKX6* | *BrCKX8* | IV | WGD | 0.3054 |
| *BrCKX8* | *BrCKX12* | IV | WGD | 0.8187 |
| *GmCKX13* | *GmCKX14* | IV | WGD | 0.7640 |
| *GmCKX5* | *GmCKX10* | IV | WGD | 0.6647 |
| *GmCKX6* | *GmCKX14* | IV | WGD | 0.7858 |
| *GmCKX6* | *GmCKX13* | IV | WGD | 0.0986 |
| *MtCKX3* | *MtCKX9* | IV | WGD | 1.0661 |
| *NnCKX4* | *NnCKX8* | IV | WGD | 0.5496 |
| *OsCKX7* | *OsCKX10* | IV | WGD | 1.3377 |
| *GmCKX2* | *GmCKX4* | V | WGD | 0.0986 |
| *MdCKX4* | *MdCKX6* | V | WGD | 0.1882 |
| *NnCKX2* | *NnCKX6* | V | WGD | 0.5072 |
| *PtCKX2* | *PtCKX4* | V | WGD | 0.3161 |
| *ZmCKX4* | *ZmCKX4b* | V | WGD | 0.1056 |
| *NnCKX5* | *NnCKX7* | VI | WGD | 0.4032 |
| *OsCKX4* | *OsCKX9* | VI | WGD | 1.1183 |
| *ZmCKX2* | *ZmCKX3* | VI | WGD | 0.1411 |
| *GmCKX7* | *GmCKX9* | VI1 | WGD | 0.1331 |
| *GmCKX7* | *GmCKX8* | VI1 | WGD | 0.5510 |
| *GmCKX8* | *GmCKX9* | VI1 | WGD | 0.6398 |
| *PtCKX1* | *PtCKX3* | VI1 | WGD | 0.1466 |
| *ClCKX2* | *ClCKX7* | VI1/VI2 | WGD | 1.4597 |
| *BrCKX3* | *BrCKX4* | VI2 | WGD | 0.4406 |
| *BrCKX3* | *BrCKX5* | VI2 | WGD | 0.3769 |
| *BrCKX4* | *BrCKX5* | VI2 | WGD | 0.4220 |
| *GmCKX1* | *GmCKX17* | VI2 | WGD | 0.0946 |
| *PtCKX5* | *PtCKX9* | VI2 | WGD | 0.2306 |
| *SlCKX2* | *SlCKX7* | VI2 | WGD | 0.8322 |

**Table S8.** Ratios of *CKX* to *IPT* gene numbers in the sampled angiosperms indicate dosage balance between *CKX* and *IPT* genes.

| Group | Species | Gene number | |  | Ratio |
| --- | --- | --- | --- | --- | --- |
|  |  | *CKX* | *IPT* |  | *CKX*/*IPT* |
| Angiosperms | *Arabidopsis thaliana*^a^ | 7 | 9 |  | 0.78 |
|  | *Brassica rapa*^a^ | 12 | 13 |  | 0.92 |
|  | *Citrus clementina* | 7 | 7 |  | 1 |
|  | *Populus trichocarpa*^a^ | 9 | 9 |  | 1 |
|  | *Glycine max*^a^ | 17 | 14 |  | 1.2 |
|  | *Fragaria vesca* | 8 | 7 |  | 1.1 |
|  | *Malus x domestica*^a^ | 12 | 12 |  | 1 |
|  | *Vitis vinifera* | 10 | 7 |  | 1.4 |
|  | *Solanum lycopersicum*^a^ | 9 | 15 |  | 0.6 |
|  | *Nelumbo nucifera*^a^ | 9 | 8 |  | 1.1 |
|  | *Zea mays*^a^ | 13 | 11 |  | 1. 2 |
|  | *Oryza sativa*^a^ | 11 | 10 |  | 1.1 |
|  | *Amborella trichopoda* | 5 | 5 |  | 1 |

^a^The monocot or eudicot species that has undergone lineage-specific polyploidization event(s).

**Table S9.** Primer sequences used for qRT-PCR amplification.

| Gene Name | TAIR ID | Forward primer (5’ to 3’) | Reverse primer (5’ to 3’) |
| --- | --- | --- | --- |
| *Tub8* | AT5G23860 | TGAGCTTATCGATTCCGTTCTC | AGATCCAGTTCCTCCTCCTAAT |
| *RD22BP1* | AT1G32640 | TATATTCTGGCAACCGTCGTATG | TATCCGTCACCTCCTCATCAAC |
| *NCED3* | AT3G14440 | CGAGCCGTGGCCTAAAGTCT | GCTCCGATGAATGTACCGTGAA |
| *CKX1* | AT2G41510 | GATTCCTAAGAGCAGCATATACC | GGAGAAAGGCTACGAGATAGAAT |
| *CKX2* | AT2G19500 | ACGTGGCCTACTTCGATTTC | GTAGAGGTTAAGCCAAGGATGAG |
| *CKX3* | AT5G56970 | TCTCAATACACAGTCAACGAGGA | TCGTACATAAACCCTCTTACATGG |
| *CKX4* | AT4G29740 | GCTTCGTAGACACCTCTTTCTT | GGAAGGGTGGTTCTGTCATAAT |
| *CKX5* | AT1G75450 | CCATGGTCCTCAAATTAGTAACG | TCTGAGCATCTCATCACCTCTC |
| *CKX6* | AT3G63440 | TGATGGAAGGACTCTGTATTGT | CCTGTCCAAGAATGCTTCATAT |
| *CKX7* | [AT5G21482](http://www.arabidopsis.org/servlets/TairObject?type=locus&name=AT5G21482) | GTAAGTCAGAAGAACGAGTCATC | AATCGGAGTCTCTGTAGTGAAG |

The primers for *NCED3* were designed according to Yu et al.^5^.

**References**

1. Matsui, A. et al. *Arabidopsis* transcriptome analysis under drought, cold, high-salinity and ABA treatment conditions using a tiling array. *Plant Cell Physiol.* **49**, 1135-1149 (2008).
2. Vyroubalová, Š. et al. Characterization of new maize genes putatively involved in cytokinin metabolism and their expression during osmotic stress in relation to cytokinin levels. *Plant Physiol.* **151**, 433-447 (2009).
3. Darwish, O. et al. SGR: an online genomic resource for the woodland strawberry. *BMC Plant Biol.* **13**, e223 (2013).
4. Jiang, Y. et al. Evolution and expression patterns of cytokinin oxidase genes in *Fragaria vesca*. *Sci. Hortic.* **212**, 115-125 (2016).
5. Yu, H. et al. Activated expression of an *Arabidopsis* HD-START protein confers drought tolerance with improved root system and reduced stomatal density. *Plant Cell* **20**, 1134-1151 (2008).
